# Cancer metabolism and intervention therapy

**DOI:** 10.1186/s43556-020-00012-1

**Published:** 2021-02-20

**Authors:** Huakan Zhao, Yongsheng Li

**Affiliations:** Department of Medical Oncology, Chongqing University Cancer Hospital, Chongqing, 400030 China

**Keywords:** Cancer, Metabolic reprogramming, Heterogeneity, Tumor microenvironment, Targeted therapy

## Abstract

Metabolic reprogramming with heterogeneity is a hallmark of cancer and is at the basis of malignant behaviors. It supports the proliferation and metastasis of tumor cells according to the low nutrition and hypoxic microenvironment. Tumor cells frantically grab energy sources (such as glucose, fatty acids, and glutamine) from different pathways to produce a variety of biomass to meet their material needs *via* enhanced synthetic pathways, including aerobic glycolysis, glutaminolysis, fatty acid synthesis (FAS), and pentose phosphate pathway (PPP). To survive from stress conditions (*e.g*., metastasis, irradiation, or chemotherapy), tumor cells have to reprogram their metabolism from biomass production towards the generation of abundant adenosine triphosphate (ATP) and antioxidants. In addition, cancer cells remodel the microenvironment through metabolites, promoting an immunosuppressive microenvironment. Herein, we discuss how the metabolism is reprogrammed in cancer cells and how the tumor microenvironment is educated *via* the metabolic products. We also highlight potential metabolic targets for cancer therapies.

## Introduction

Although multiple strategies for cancer treatment have been applied in clinics, cancer remains one of the leading causes of mortality worldwide, accounting for 13 % of all deaths [1]. Overwhelming evidence manifests that resistance to therapy is becoming the biggest challenge for the successful treatment of cancer [2], and severe side effects have restricted the application of some cancer-therapies. For example, the intrinsic or acquired resistance to chemotherapy or radiotherapy leads to relapse and metastatic progression of the tumor [3]. Besides, immunotherapies such as immune checkpoint inhibitors (ICB) and chimeric antigen receptor T cells (CAR-T) have been approved for application for several malignancies. However, a majority of patients with solid tumors couldn’t benefit from these treatments, and the side effects, including neurologic toxicity and the “cytokine storm”, have impeded their full application to cancer therapy. Therefore, developing anti-cancer strategies with high efficacy and low side effects is critical for cancer treatment.

Massive efforts have been attempted to improve the effects of tumor treatments; for example, benefits of approaches by the elimination of cancer stem cells (CSCs) and anti-inflammation have been proved in clinics or preclinical models [4, 5]. In recent years, observations suggest cancer metabolism contributes to carcinogenesis and cancer progression and correlates with outcomes of cancer patients [6]. The emerging view is that the genetic mutations and activation of oncogenic pathways are integrated into downstream metabolic reprogramming, which orchestrating various malignant activities, including proliferating, metastasis, and survival under stress conditions [7]. Moreover, metabolic heterogeneity is much less than genetic heterogeneity in tumor cells [8]. Over the past few decades, with advances in the understanding of tumor metabolism, reprogrammed metabolic activities of cancer cells have been exploited to diagnose, monitor, and specific cancer therapy. For example, the character of glucose uptake by cancer cells has been successfully explored in clinics through the application of fluorodeoxyglucose positron emission tomography (FDG/PET) imaging to monitor cancers and assess response to therapy [9]; statins, specific hydroxy-3-methyl glutaryl coenzyme A reductase (HMGCR) inhibitors, significantly reduce the risk of prostate and breast cancer, and inhibit the progression of certain cancers [10]. Although many metabolic regulators failed to be adopted in clinics due to ineffective or toxic side effects *in vivo*, targeting cancer metabolism remains a promising therapeutic approach for cancers.

Tumors reprogram multiple pathways associated with nutrient acquisition and metabolism to fulfill the biosynthetic, bioenergetic, and redox demands of malignant cells. For example, tumor cells mobilize various intracellular anabolic pathways to provide abundant biosynthetic precursors such as nucleotides, proteins, and lipids, as well as fulfill the energy demand for producing adenosine triphosphate (ATP) for rapid proliferation [11, 12]. However, catabolic metabolism (*e.g.,* OXPHOS, FAO, autophagy) is the primary metabolic characteristic of metastatic cancer cells, which always suffer from metabolic stress [13]. Furthermore, tumor metabolic heterogeneity and plasticity endow tumor cells with abilities of resistance to various therapies [14, 8]. Hence, a comprehensive understanding of the mechanisms of metabolic reprogramming will contribute to developing better anti-cancer strategies. In this review, we discuss the mechanism of metabolism reprogramming in cancer cells, and biofunctions of metabolites in the TME, as well as suggest potential metabolic targets for cancer therapies.

## The primarily anabolic pathways in cancer cells

**Fig. 1:**
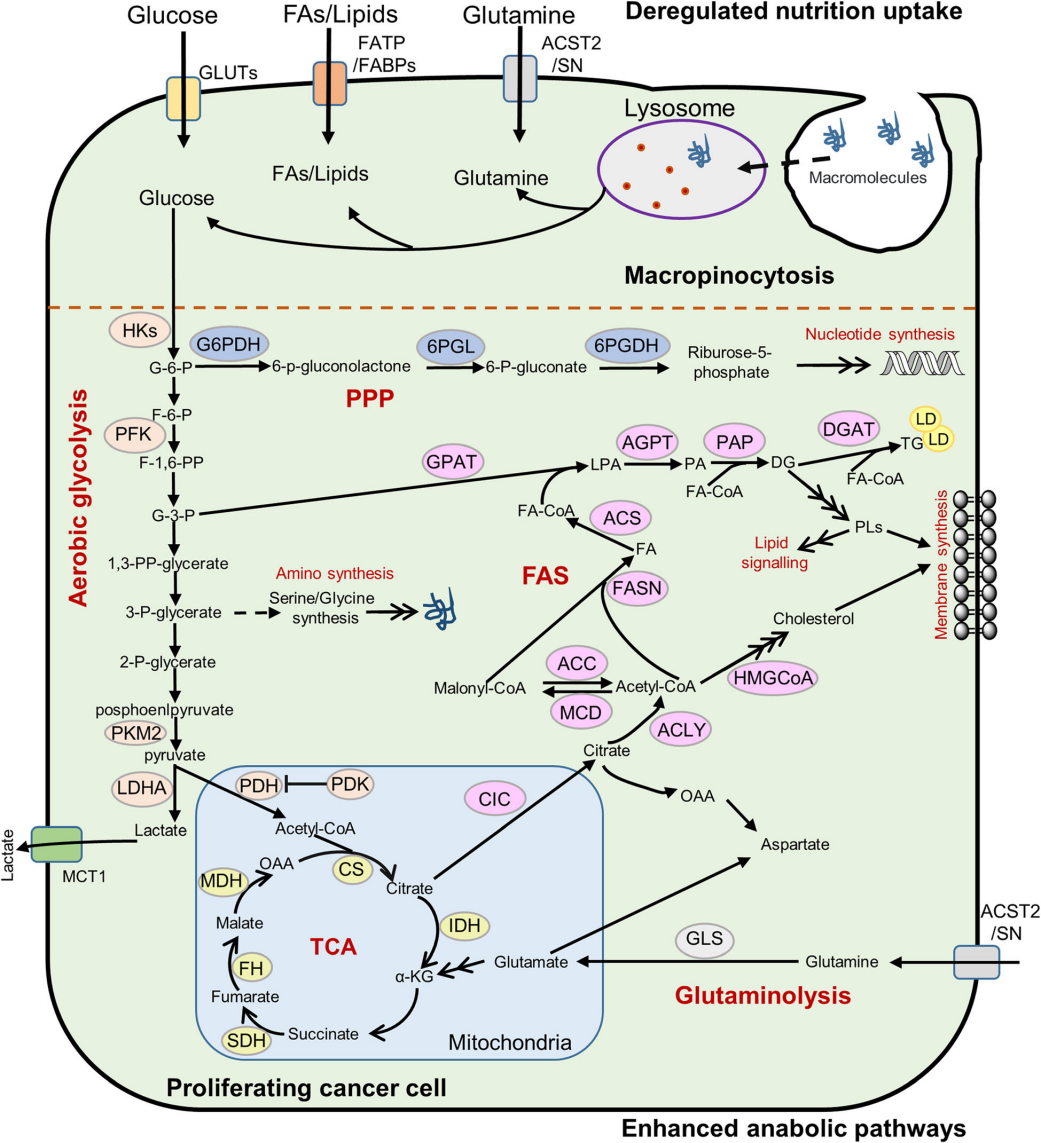
The primarily anabolic pathways of cancer cells. Tumor mobilize various nutrients uptake and intracellular anabolic pathways to provide abundant cellular building blocks such as nucleic acid, protein and lipid for rapid proliferation. Cancer cells obtain glucose, glutamine and fatty acids *via* transport proteins, respectively; certain cancers acquire mutations that can capture extracellular macromolecules through macropinocytosis. Then, the nutrients obtained from extracellular enter multiple anabolic pathways including rapid aerobic glycolysis, glutaminolysis, *de novo* FAS and nucleotide synthesis to fulfill the biosynthetic and energetic demands of rapid proliferation

Compared with normal cells, which rely primarily on oxidative phosphorylation (OXPHOS) to generate the energy needed for cellular processes, most tumor cells show significantly enhanced anabolism pathway, including aerobic glycolysis, glutaminolysis, fatty acid synthesis (FAS), and pentose phosphate pathway (PPP) (Fig. 1). Aerobic glycolysis provides sufficient intermediate metabolites such as glucose-6-phosphate (G-6-P) and glyceraldehyde-3-phosphate (G-3-P) for multiple biosynthetic pathways [15, 11]; the *de novo* FAS can meet the requirements of phosphatidylethanolamine (PE), phosphatidylcholine (PC), and cholesterol in the cell membrane synthesis [16]. Besides, intermediates of the tricarboxylic acid (TCA) cycle, such as citrate, oxaloacetic acid, α-ketoglutarate (α-KG), are utilized in the synthesis of fatty acids (FAs) and non-essential amino acids (NEAAs) [17, 18]. In addition, glutamine produces glutamate under the action of glutaminase, which is converted to α-KG, thus replenish the TCA cycle and maintain its balance [19]. Furthermore, in addition to generating phosphopentoses and ribonucleotides, the PPP is a primary source of NADPH, which is an important antioxidant for cellular redox adaption [20–22].

### Dysregulated uptake of nutrition

In order to fulfill the biosynthetic and energetic demands of rapid proliferation, tumor cells must uptake nutrients from a frequently nutrient-poor environment. Cancer cells devouringly absorb glucose, glutamine, and FAs, which are catabolized and utilized as building blocks for various macromolecules [23–25]. Certain cancers also employ alternative ways of uptake necessary nutrients when amino acids are insufficient, for instance, macropinocytosis, entosis of living cells, and phagocytosis of apoptotic bodies [26, 27]. Noteworthily, different cancers (or subtypes) always possess distinct metabolic requirements for nutrients (Table 1).

**Table 1:** Different tumors with distinct metabolic requirements

Cancer type	preferences for nutrients	The principal metabolic pathway
Breast cancer (ER+)	Lactate from CAFs (metabolic coupling)	OXPHOS
Breast cancer (TNBC)	Glucose	Glycolysis
Prostate cancer (Early-stage)	Lactate from CAFs (metabolic coupling)	OXPHOS
Prostate cancer (Late-stage)	Glucose and glutamine	Glycolysis
Metatatic Prostate cancer cells	Free fatty acids	FAO
Melanoma	Free fatty acids	FAO
Hepatocellular carcinoma (without β-catenin mutant)	Glucose and glutamine	Glycolysis
Hepatocellular carcinoma (with β-catenin mutant)	Free fatty acids	FAO
Colorectal cancer	Glucose and glutamine	Glycolysis
Fast-cycling glioblastoma cells	Glucose and glutamine	Glycolysis
Slow-cycling glioblastoma cells	Free fatty acids	FAO
Ovarian cancer	Glucose and glutamine	Glycolysis
Ovarian cancer cells with peritoneal metastasis	Free fatty acids	FAO

As the most important energy in malignance, glucose not only be used to generate ATP through glycolysis and OXPHOS but also provide precursors of amino acids, nucleotides, and lipids [28, 29]. The first rate-limiting step of glucose metabolism is the transportation of glucose into the cytoplasm, which is mediated by glucose transporters (GLUTs). GLUT1-4, particularly GLUT1/3, are often aberrantly up-regulated in different cancer types and significantly strengthen the glucose metabolic flow [30, 31]. For instance, high expression of GLUT1/3 is associated with poor survival and tumor progression in many types of cancer, including lung cancer, hepatocellular carcinoma (HCC), colorectal cancer (CRC), and ovarian cancer (OVC). Additionally, inhibition of glucose transporters by phloretin and glucocorticoids can obviously sensitize cancer cells to chemotherapy [32, 33]. Furthermore, the application of ^18^F-fluoro-2-deoxyglucose positron emission tomography (^18^F-FDG/PET), based on the property of enhanced glucose uptake in cancer cells, is widely applied in the diagnosis and monitoring of some types of cancers [9].

Glutamine is a major energy substrate which supplies carbon and reduces nitrogen for the biosynthesis of purine, glucosamine-6-phosphate, pyrimidine nucleotides, and NEAAs [19]. Most proliferating tumor cells rely on a continuous supply of glutamine to maintain the integrity of TCA cycle intermediates. Moreover, in many cancer cells, glutamine is required for support of the NADPH production needed for redox homeostasis and macromolecular synthesis [34]. Glutamine is transported across the cell membrane by three amino acid-transporters: sodium-neutral amino acid transporters, alanine, serine, cysteine–preferring transporter 2 (ASCT2); and large neutral amino acid transporter 1 (LAT1) [19]. ASCT2, the most prominent glutamine transporter, is remarkably upregulated in many cancer cells, and high expression of ASCT2 is positively correlated with poor prognosis in tumor patients [35]. Moreover, suppressing the glutamine uptake by inhibiting ASCT2 can suppress the growth of tumor cells both *in vitro* and *vivo* [36, 37]. Multiple types of cancer cells, such as pancreatic cancer cells, glioma cells, and lung cancer cells, are sensitive to glutamine deprivation [34], indicating that targeting glutamine uptake is now a potential therapeutics. Although ^18^F-FDG/PET has been successfully applied for diagnosis and monitoring therapeutic effect of tumors in clinics [9], a number of malignant tumors are ^18^F-FDG/PET-negative. Furthermore, the application of ^18^F-FDG/PET to monitor tumors in certain tissues (*e.g.,* brain) with high ability of glucose uptake will cause a strong background, making diagnosis inaccuracy. As an alternative metabolic tracer, ^18^F-(2S, 4R)4-fluoroglutamine has been developed as a PET tracer for mapping glutaminolytic tumors, which may further assist the diagnostic capacity of ^18^F-FDG/FET for cancer patients and evaluate the metabolic changes in certain tumors [38].

Cancer cells frequently display dysregulated fatty acid metabolism to provide metabolites for anabolic processes and meet energy requirements [39, 40]. Some tumors scavenge free FAs from their environment* via *the up-regulation of fatty acid transporters, including the fatty acid transport proteins (FATPs) [41] and the fatty acid-binding proteins (FABPs) [42]. For example, FABP4 is involved in transporting FAs from surrounding adipocytes for OVC [43]; abnormally over-expressed FATP1 mediates FAs uptake in human melanomas, and inhibition of FATP1 by lipofermata not only decreases melanoma lipid uptake but also reduces invasion and growth [44]; CD36, a widely expressed transmembrane protein with diverse functions that include fatty acid uptake, has been implicated in breast cancer, HCC, OVC, etc. [45, 46]. These data demonstrate that FA uptake pathways as a potential target for certain types of tumors. Besides, cholesterol synthesis is an ATP- and NADPH consuming multistep reaction, while glioblastoma cells can directly uptake the cholesterol secreted by neighboring normal astrocytes, saving the cost of cholesterol synthesis, thus possessing a growth advantage [47, 48].

In spite of the avarice with which they take up low-molecular-weight nutrients, cancer cells *in vivo* often confront conditions of nutrient scarcity because of the inadequate tumor vascular supply [49]. Certain cancers acquire mutations that can activate the ability to capture extracellular macromolecules. For instance, when free amino acids are unavailable, cancer cells with mutant Ras or c-Src alleles can recover amino acids from extracellular proteins *via* macropinocytosis, entosis of living cells, or phagocytosis of apoptotic bodies [26]. Macropinosomes are trafficked into the cytoplasm, where they fuse with lysosomes, and the engulfed proteins are proteolytically degraded to liberate free amino acids [27]. In short, tumor cells use opportunistic modes of nutrient obtaining to survive and proliferate in a nutrient-poor condition.

### Anaerobic glycolysis

Most differentiated cells preferentially utilize glucose to generate ATP with high efficiency, which firstly converts glucose to pyruvate through glycolysis, and then metabolizes pyruvate *via* the TCA cycle and subsequent OXPHOS [50, 11]. However, cancer cells divert glucose-derived pyruvate away from mitochondria and toward lactate production even in oxygen-rich conditions, described by Otto Warburg as aerobic glycolysis [50, 11]. Aerobic glycolysis provides several advantages for proliferating cancer cells [11, 29]. First, glucose fermentation supplies cells with various intermediates for biosynthetic pathways. For instance, glycerol and acetyl-CoA are produced for lipid biosynthesis, and ribose is generated for the synthesis of nucleotides. NEAAs and NADPH redirect carbohydrates to biosynthetic pathways. Second, although the production of ATP per glucose consumed by uncomplete oxidation in aerobic glycolysis is much lower than OXPHOS (2 *vs*. 36 ATP), the high rate of ATP production during glycolysis due to enhanced glycolytic flux, the percentage of ATP produced from aerobic glycolysis can exceed that produced from OXPHOS in most proliferating cells. Third, high levels of lactate generated by aerobic glycolysis correlate with increased metastasis, recurrence, and poor outcome of cancer patients. The increased anaerobic glycolysis of tumor cells mainly due to the up-regulated expression or activity of key enzymes involved in glycolysis, such as hexokinase 2 (HK2), phosphofructosase kinase (PFK), pyruvate kinase M2 (PKM2), pyruvate dehydrogenase kinase (PDK), lactate dehydrogenase (LDH) and other key glycolytic enzymes [11, 51].

HK2, the enzyme which catalyzes the first committed step of glycolysis, phosphorylates glucose to generate glucose-6-phosphate (G-6-P) [52]. With ATP consumption, the reaction mediated by HK2 is highly exergonic and is considered irreversible in the cytoplasm, which preventing glucose efflux back into the extracellular space. Moreover, HK2 has been considered as a key cancer biomarker, which is associated with an advanced state of tumors and is related to poor prognosis of patients [53]. Knockdown of HK2 causes a substantial decrease in glycolysis, and decreased proliferation in cancer cells, suggesting that targeting HK2 is a potential strategy for cancer therapy [53]. 2-deoxyglucose (2-DG), a glucose analog competitively inhibits glucose phosphorylation, showed good toleration in combination with radiation therapy in a clinical trial of glioblastoma multiforme [54]. Nowadays, the unsuccessful clinical application of HKs inhibitors is partially due to low selectivity for specific isoforms and excessive side effects.

Conversion of fructose-6-phosphate (F-6-P) to fructose-1,6-bisphosphate (F-1,6-BP), the second committed step of glycolysis, is catalyzed by PFK [55]. PFK1 is a tetrameric protein consist of PFK-M (muscle), PFK-L (liver), and PFK-P (platelet), and the main isoenzymes expressed in tumor cells are PFK-P and PFK-L. Besides, in response to proliferative signals, the PFK1 activity is increased in cancer cells to provide abundant glycolytic flux and coordination of glucose entry into glycolysis [56, 55]. For instance, PFK1 activity is activated by hypoxia-inducible factor-1A (HIF-1α) or the oncogene Ras [55]; the transcription repressor Snail1 switches the glucose flux towards PPP by repressing PFK-P, generating NADPH with increased oxidative PPP, thus promoting breast cancer cell survival under metabolic stress [57]. Thus, inhibiting PFK directly in cancer cells is not reasonable since it is also crucial to glycolysis in normal cells, as well as suppression of PFKP reprograms glucose metabolism from aerobic glycolysis towards PPP.

Pyruvate kinase catalyzes the last irreversible step of glycolysis, in which a high-energy phosphate group is transferred from phosphoenolpyruvate (PEP) to adenosine diphosphate (ADP) to produce pyruvate and ATP [58]. There are four mammalian isomers of PK: PKL, PKR, PKM1, and PKM2, which are tissue-specific. PKL is the major isoform in the liver, intestine, and kidney, whereas PKR is predominantly expressed in erythrocytes. PKM1 and PKM2 are encoded by the PKM gene *via* alternative splicing of mutually exclusive exons: PKM1 only contains exon 9 while PKM2 exclusively contains exon 10 [59]. However, substantial sequence similarity, PKM1, and PKM2 display diverse catalytic and regulatory properties. PKM1, expressed in tissues that require a massive supply of ATP such as muscle and brain, possess constitutively high catalytic activity [59], while PKM2 is predominantly up-regulated in highly proliferative tumor cells with growing anabolic demands [60, 61]. In addition, PKM2 enzyme activity is subjected to allosteric regulation through stabilization or destabilization of the enzyme tetramer. Several investigations have reported that knockdown of PKM2 leads to decreased metabolic activity, reduced tumorigenesis, and the death of cancer cells. On the contrary, quinolone sulfonamide, a PKM2 activator, promotes the conversion of PKM2 from dimer to tetramer, resulting in an attenuated synthesis of the glycolytic intermediates used as biosynthetic precursors in cancer cells [62, 61]. Moreover, PKM2 activators also suppress the growth of xenograft tumors such as aggressive lung adenocarcinoma. Nowadays, several PKM2 activators and inhibitors are in preclinical and clinical trials, and the results suggest that PKM2 is a promising target for cancer treatment [63, 64].

The mitochondrial pyruvate dehydrogenase complex (PDHs) decarboxylates pyruvate to generate acetyl-CoA, which is a critical step from the glycolytic pathway to TCA [28]. PDK is the enzyme that phosphorylates PDHs, leading to the inactivation of PDHs. Elevated expression of PDKs induces a “glycolytic shift” towards glycolysis instead of OXPHOS [11, 29]. Therefore, PDKs are regarded as a pivotal "regulator" of pyruvate entry into TCA. PDKs, particularly PDK1 and 4, are frequently overexpressed in diverse cancers and frequently related to cellular proliferation, drug resistance, invasion, and metastasis [65]. Multiple transcription factors, including Myc, Wnt, and HIFs, act alone or synergistically to transcriptionally activate expressions of PDKs [66]. Evidence suggests that inhibition of PDKs can reverse the “Warburg effect,” reduce lactate concentration and enhance OXPHOS in tumor cells. Recently, scientists have yielded several small-molecule PDK inhibitors [66, 65]; for instance, 2-chloroacetic acid (DCA) can shift cellular metabolism from glycolysis to OXPHOS, exhibiting the potential effect of antitumor action [67]. Despite theoretically possible, recently synthesized PDK inhibitors remain far from their clinical applications.

Lactate dehydrogenase A (LDHA), mainly located in the cytoplasm, facilitates the glycolytic process by converting pyruvate to lactate and transforming NADH to NAD^+^ [68]. Aberrant expression of LDHA is a hallmark of multiple cancers, including CRC, HCC, esophageal squamous cell carcinoma (ESCC), squamous head and neck cancer, and non-small cell lung cancer (NSCLC) [69, 70]. In cancer cells, LDHA could be regulated by numerous transcription factors, including c-Myc, HIF-1, cAMP response element-binding protein (CREB), forkhead box M1 (FOXM1) and krüppel-like factor 4 (KLF4), etc. Tumor cells with reduced LDHA activity failed to maintain high ATP levels, which likely contribute to the suppression of cell proliferation [71]. For instance, LDHA knockdown suppresses tumor growth and tumorigenicity in ESCC or k-Ras-induced NSCLC mouse model, and LDHA-knockdown HCC cells show increased apoptosis; and inhibition of LDHA by FX11 dramatically reduces ATP levels, then induces significant oxidative stress and inhibits the progression of human lymphoma as well as pancreatic cancer xenografts [71]. In addition, cell lines dependent on the PPP and glycolysis are sensitive to LDHA inhibitors [71]. However, cancer cells which depend on FAS and glutamine decomposition are unaffected by LDHA inhibitors, since these cells rely on a mitochondrial function to produce ATP once the production of lactate is blocked [70]. Hence, LDHA is considered as a potential target for the prevention and treatment of cancers which depend on glycolysis and PPP.

Taken together, the “Warburg effect” prominently benefits both bioenergetics and biosynthesis in cancer cells [11]. Suppression of glycolysis significantly inhibits proliferation and metastasis in cancer cells, as well as makes tumor cells susceptible or sensitive to therapeutic intervention [29, 28, 11]. Recently, several preclinical investigations have demonstrated the effectiveness of this therapeutic approach. Therefore, targeting anaerobic glycolysis is attractive for therapeutic intervention.

### Truncated TCA cycle

As the biosynthetic and bioenergetic organelles of cells, mitochondria take up substrates from the cytoplasm and utilize them to drive the TCA cycle, fatty acid oxidation (FAO), the electron transportation, and OXPHOS-the basic processes of ATP production, and to synthesize nucleotides, amino acids, lipids, and NADPH [17, 18]. When normal mitochondria are transferred into the cytoplasm of tumor cells, the tumorigenic phenotype is suppressed [72]. Furthermore, the tumorigenic phenotype is remarkably enhanced when tumor mitochondria are injected into a normal cell cytoplasm [72]. Accumulating evidence indicate that the disturbed function of mitochondrion plays a pivotal role in the tumorigenesis and progression of various cancers [73, 74].

The TCA cycle is a central hub for OXPHOS in cells and fulfills their bioenergetic, biosynthetic, and redox balance requirements [11]. When oxygen is abundant, normal cells first metabolize glucose to pyruvate *via* glycolysis and then completely oxidize most pyruvate to CO_2_ through the TCA cycle to maximize ATP production. Nevertheless, cancer cells can redirect the pyruvate generated by glycolysis away from TCA by generating lactate, resulting in minimal ATP production compared with OXPHOS [75]. On the one hand, high expression of PDKs negatively regulates the PDHs and inhibits the conversion of pyruvate to acetyl-CoA, which is important in transferring pyruvate into the TCA cycle but not into lactate production [65]. On the other hand, mutations or deregulated expression in the TCA cycle enzymes leading to a truncated TCA cycle, resulting in defected OXPHOS and enhanced anaerobic glycolysis, as well as epigenetic changes that are correlated with progression of cancer [76]. Some tumors harbor mutations of TCA enzymes, such as isocitrate dehydrogenase (IDH), succinate dehydrogenase (SDH), fumarate hydratase (FH), and malate dehydrogenase (MDH). These mutations lead to metabolic shifts, which contribute to the progression of cancers [76].

The IDH family (IDH1, IDH2, and IDH3) are mainly known for their role in catalyzing the conversion of isocitrate to α-KG [77]. IDH1 is expressed in the cytoplasm and peroxisomes, while IDH2 and IDH3 are located in the mitochondrial. IDH1 and IDH2 couple the interconversion between cytosolic isocitrate and α-KG in a NADP^+^/NADPH-dependent reaction, while IDH3 can only oxidize isocitrate to α-KG and requires NAD^+^ as the co-factor [77, 78]. Missense mutations in IDH1/2 occur primarily at the active arginine residues that play a key role in isocitrate binding, while no mutations in IDH3 have been reported. In clinical studies, IDH1/2 mutations were observed in low-grade glioma and secondary glioblastoma (∼80%), and in acute myeloid leukemia (∼20%), angioimmunoblastic T-cell lymphomas (16%–17%) with worse prognosis [79–81, 77]. Mutations in IDH1/2 lead to elevated levels of 2-hydroxyglutarate (R-2-HG or D-2-HG), which is a competitive inhibitor of oxoglutarate-dependent dioxygenases and an oncogenic factor for promoting leukemogenesis [79]. Besides, increased production of D-2HG in IDH-mutant tumors interferes with the activity of several a-KG-dependent dioxygenases, including the prolyl hydroxylases that degrade HIF-1α protein. In addition to D-2HG, several other TCA cycle metabolites, including a-KG, succinate, and acetyl-CoA, have emerged as mediators of early tumorigenesis [79]. Alternatively, such mutations may contribute to the production of citrate from α-KG as a carbon precursor for macromolecular synthesis. Moreover, specific inhibitors targeting mutant IDH can revert glioma cells to a more differentiated state [82, 79]. AG-221, a first-in-class therapy targeting acute myeloid leukemia (AML) harboring oncogenic IDH2 mutations, suppress the 2-HG production and induce cellular differentiation in primary human IDH2 mutation-positive AML cells *in vitro* and *vivo* [83].

SDH, also known as complex II, is an enzyme located in the inner mitochondrial membrane, where it oxidizes succinate to fumarate in the TCA cycle and reduces ubiquinone to ubiquinol in the electron transport chain (ETC) [84]. The SDH complex consists of four subunits (SDHA, SDHB, SDHC, and SDHD), and is regarded as a tumor suppressor because a deficiency of this enzyme is known to activate tumorigenesis *via* dysregulation of HIF activity [85]. Additionally, mutations or deletion of SDH have been observed in gastrointestinal stromal tumors (GIST), renal tumors, thyroid tumors, neuroblastoma, and testicular seminoma, hereditary paragangliomas (hPGLs), OVC, and pheochromocytomas (PCCs), implicating its importance in multiple cancers [85]. Furthermore, deficiency of the enzyme results in the accumulation of succinate, identified as an “oncometabolite,” is causing non-metabolic effects that promote the transformation in receptive contexts, such as regulating the methylation status of histones and DNA [86, 87]. Besides, decitabine, a DNA methylation inhibitor, can suppresses the proliferative and invasive phenotype of SDH-deficient cells [88], indicating that epigenetic silencing induced by succinate accumulation is associated with malignant behaviors in SDH-mutated tumors.

FH exists in cytosolic and mitochondrial forms and is a homotetrameric cycle enzyme that catalyzes the stereospecific and reversible hydration of fumarate to malate [72, 85]. Beyond its mitochondrial role, FH expressed in cytoplasm participates in the urea cycle, nucleotide and amino acid metabolism. Studies show that FH has been proposed to function as a tumor suppressor, as well as mutations, including missense, frameshift, nonsense, and large deletions exists in FH identified in the breast, bladder, and testicular cancer [84, 85]. In addition, heterozygous mutations in FH predispose patients to uterine leiomyomas (MCUL), hereditary leiomyomatosis, and renal cell cancer (HLRCC) [76]. Loss of FH cause striking metabolic changes that are required to compensate for the truncation of the TCA cycle and for the abnormal accumulation of fumarate [84]. Firstly, fumarate can accumulate to millimolar levels in FH-deficient tumor cells, and high fumarate levels can alter multiple enzymatic reactions in which fumarate is directly involved as either a substrate or product. Similarly to succinate, an increase in fumarate also inhibits the prolyl-hydroxylases, which contribute to the degradation of HIF-1α protein [89, 90]. Additionally, fumarate upregulation may also cause post-translational modification and inactivation of Kelch-like ECH-associated protein 1 (Keap1), then activate nuclear factor erythroid 2-related factor (Nrf2), the key regulator of cellular antioxidant. Another target of fumarate accumulation is the superfamily of α-KG dependent dioxygenases (aKGDDs), which is involved in protein hydroxylation, DNA and histone demethylation, and RNA modifications [89]. Of note, succinate is identified as an “oncometabolite,” an epigenetic modulator, an important regulator of ROS metabolism and posttranslational modifications.

There are two main isoenzymes of malate dehydrogenase (MDH): The cytoplasmic MDH1 and the mitochondrial MDH2 [18]. Mitochondrial MDH2 is responsible for the reversible oxidation of malate to oxaloacetate (OAA) through NAD+ to NADH reduction in the ETC, while cytosolic MDH1 catalyzes the conversion of OAA to malate with reduction of NAD+ to NADH, forming the malate/aspartate shuttle [74]. Compared to normal counterparts, both the MDH1 and the MDH2 enzymes display elevated levels in NSCLC, while only high expression of MDH1 is related to the poor prognosis of NSCLC patients. Furthermore, knockout of MDH1 leads to significantly higher toxicity than depletion of MDH2, indicating that MDH1 as a potential therapeutic target in these tumors [91]. In spite of mutations in the MDH2 gene are observed in several cancers, including uterine cancer, prostate cancer, pheochromocytoma, and other paragangliomas, MDH2 overexpression was observed in doxorubicin-resistant uterine and prostate cancer cells and may contribute to drug resistance in disease models [18]. Furthermore, MDH2 inhibition by small-molecule compound 7 leads to a decrease of mitochondrial respiration by the reduction of NADH levels [92], implying that MDH2 is a potential target in cancer therapeutics due to its effect on ATP production and chemotherapy resistance.

Beyond mutations observed for TCA cycle enzymes, several studies have demonstrated that other TCA cycle-related enzymes, including citrate synthase (CS), aconitate hydratase (AH), are dysregulated in tumors [18, 74]. CS catalyzes the synthesis of citrate from OAA and acetyl-CoA, which is a rate-limiting step in the TCA cycle. In addition, overexpressed or enhanced enzymatic activity of CS in NSCLC, OVC, pancreatic and renal cancer is associated with poor prognosis [93]. Besides, AH catalyzes the conversion of citrate to isocitrate, and its expression is downregulated in gastric and prostate cancer [94].

In general, multiple types of cancer are marked by TCA cycle dysfunction, which results in defected OXPHOS and enhanced anaerobic glycolysis, as well as epigenetic changes that are correlated with carcinogenesis and progression of cancer. As a result, certain enzymes of the TCA cycle, such as mutated IDH and decreased KGDHC, may be exploited for the therapeutic targets. Therefore, the rescue of TCA cycle dysfunction is a promising approach for cancer therapy.

### Glutaminolysis

The majority of pyruvate derived glucose is utilized to generate lactate other than entering the TCA cycle in cancer cells [11]. Many tumors utilize glutamine to replenish the TCA cycle, for macromolecules synthesis and ATP production, even re-enter the glycolytic pathway through malate or OAA. Some cancer cells actively import glutamine from extracellular fluids *via* glutamine transporter, whereas others prefer to synthesize glutamine from glutamate and ammonia by glutamine synthetase (GLS). As a preferential substitute of glucose, glutamine can fuel the TCA cycle through its conversion to α-KG by aspartate transaminase (AST) and alanine transaminase (ALT), as well as glutamate dehydrogenase (GLUD) [95, 96, 34]. Subsequently, α-KG enters TCA cycle to supply substrate, which is used for macromolecules synthesis and ATP production [11]. In many cancer cells, glutamine is also required for the maintenance of mitochondrial membrane potential and integrity and for support of the NADPH production needed for redox control [34]. Supplement with nucleotide bases can rescue the proliferation of glutamine-deprived cells, indicating that the generation of nucleotide base is important downstream of glutamine metabolic pathways [34]. Given that glutamine is an important fuel source for the TCA cycle. Thus blockage of glutaminolysis through small molecule inhibitors is an attractive therapeutic approach to these tumors addicted to glutamine [95]. Recently, GLS inhibitors such as compounds C-968, BPTES, and V-9302 shows good effects and are being tested in clinical trials at different stages [95]. These suggest that selective inhibition of glutamine metabolism (such as glutaminase or glutamine aminotransferase) might produce an anti-cancer effect.

### Pentose phosphate pathway

Nucleic acids must be duplicated during cell division. Therefore *de novo* synthesis of nucleotide is essential for tumor cell proliferation. The PPP works in parallel to glycolysis, providing ribonucleotides for DNA synthesis and NADPH [20]. The PPP consists of two branches: the oxidative branch and the non-oxidative branch [97, 20]. PPP branches from glycolysis at the first committed step of glucose metabolism is catalyzed by HKs and consumes G-6-P as a primary substrate. Subsequently, glucose-6 phosphate dehydrogenase (G6PDH), the first and rate-limiting enzyme in the oxidative branch of PPP, dehydrogenates G-6-P to yield 6-phosphogluconolactone, and generates NADPH by the reducing NADP+. After that, 6-phosphogluconolactone is hydrolyzed by 6-phosphogluconolactonase (6PGL) into 6-phosphogluconate (6PG), which is subsequently decarboxylated by 6-phosphogluconate dehydrogenase (6PGDH) to generate ribulose-5-phosphate (Ru5P) and a second NADPH. Under the activation of ribose-5-phosphate isomerase (RPI), Ru5P is then transformed into ribose-5-phosphate (R5P), which is essential for for the synthesis of ribonucleotides. Furthermore, the non-oxidative branch of PPP yields fructose-6-phosphate (F-6-P) and glyceraldehyde-3-phosphate (G-3-P) *via* a series of reversible reactions mediated by multiple enzymes such as fructose-6-phosphate (F-6-P) and glyceraldehyde-3-phosphate (G-3-P).

Active PPP flux is crucial for tumor progression, because it not only provides phosphopentoses and ribonucleotides for the high-rate synthesis of nucleotides, but also generates NADPH, which is essential for redox homeostasis during tumor growth or stress [98]. For example, aberrant activation of G6PDH is involved in proliferation, metastasis, as well as adaptation for stressful environments in multiple types of cancer, making it as a promising target for anti-cancer therapy. Besides, studies demonstrate that the elevation of the PPP facilitates resistance to anti-cancer therapies causing DNA damage or oxidative stress, such as chemotherapy and radiotherapy [99]. Recent studies indicate that robust expressions of G6PDH, hallmarks of activated oxidative PPP, are observed in drug-resistant cancer cells [97]. In addition, the nicotinamide analogue 6-aminonicotinamide (6AN), the inhibitor of the oxidative PPP, can sensitizes various cancer cell lines to cisplatin; another inhibitor of oxidative PPP-dehydroepiandrosterone (DHEA) can reverse the castration-resistant prostate cancer [100, 101]. Collectively, hyperactive PPP flux dramatically facilitates cancer growth and survival, as well as therapy resistance, suggesting that inhibition of PPP might be an attractive way to fight against cancer [22].

### *De novo* fatty acid synthesis and lipid biosynthesis

FAs are consist of a terminal carboxylic acid group and a hydrocarbon chain of varying carbon lengths and levels of desaturation; they are the main components of diverse lipids, including triglycerides (TGs), sphingolipids, sterol esters (SEs), and phospholipids (PLs) [102, 103]. Recently, dysregulated metabolic reprogramming of FAs is perceived as a significant feature of multiple cancers [104, 40]. In order to maintain rapid proliferation, tumor cells must divert carbon from energy-producing towards FAs for the biosynthesis of membranes and signaling molecules [16]. It has long been known that most normal cells prefer extracellular sources of FAs, while most FAs that make up the components of membranes come from *de novo* synthesis rather than up-taken exogenously in cancer cells [16]. Glucose metabolism feeds into FA metabolism at the point of citrate, an intermediate in the TCA cycle [11]. Several steps are required to the conversion of citrate to bioactive FAs, and these steps are catalyzed by ATP citrate lyase (ACLY), acetyl-CoA carboxylase (ACC), fatty acid synthase (FASN), and acyl-CoA synthetases (ACS) [40].

Different subcellular localization of citrate have diverse metabolic fates: mitochondrial citrate enters into the TCA cycle, while cytoplasmic citrate feeds into FAS. Citrate carrier (CIC) transports citrate across the inner mitochondrial membrane for use in the cytoplasm [105]. Furthermore, CIC expressions are increased in multiple cancer cell lines and tumors and are correlated with poor outcomes of patients [106]. Benzene-tricarboxylate analog (BTA), an inhibitor of CIC, shows significant antitumor effects in various tumor types and xenograft mice model [105]. Once transported to the cytosol, mitochondrial citrate is converted by ACLY to acetyl-CoA accompanied by ATP consumption, which is an essential biosynthetic precursor for FAS and mevalonate pathway.

Acetyl-CoA is then carboxylated by ACC to form malonyl-CoA, and ACC is the most up-regulated enzyme in the FAS pathway [107]. ACC activity is positively regulated by citrate and glutamate, while negatively regulated by fatty acyl-CoAs such as palmitoyl-CoA, and ACC phosphorylation mediated by AMP-activated protein kinase (AMPK) is enzymatic inactivated [108]. There are two ACCs in the human: ACC1 is highly enriched in lipogenic tissues such as adipose tissue, and ACC2 occurs in oxidative tissues, *e.g.*, skeletal muscle [107]. Because they primarily exist different specialized tissues, ACC1 and ACC2 play different metabolic roles: malonyl-CoA generated by ACC1 serve as a substrate for FA synthesis, whereas the malonyl-CoA made by ACC2 inhibits carnitine palmitoyltransferase (CPT1), a first and rate-limiting enzyme for β-oxidation of FA, thus preventing FA degradation [109, 107]. The knockdown of ACC1 or inhibition of ACC1 and ACC2 by soraphen-A induces apoptosis in prostate and breast cancer cells, whereas not in nonmalignant cells [110]. However, TOFA (5-(tetradecyloxy)-2-furoic acid), an allosteric inhibitor of ACC1, fails to show anti-tumor effect in breast cancer cells; and silencing of ACC1 or ACC2 boosts the lung cancer growth by promoting NADPH-dependent redox balance [111]. Furthermore, there is experimental evidence show that AMPK activator, such as metformin, has antitumor activity *in vitro* and *in vivo* in mice and humans [112]. Taken together, the role of ACC in cancer cells remains further explored.

FASN, the most studied lipogenic enzyme in cancer, is responsible for the production of palmitate (C16:0) from acetyl-CoA and malonyl-CoA substrates in the presence of NADPH [16]. FASN is frequently upregulated in a multitude of cancers and is strongly correlated with a poor prognosis in many instances, as well as associated with chemoresistance and metastasis [16]. Nowadays, FASN is an attractive therapeutic target because most cancer cells depend upon FASN-mediated *de novo* FAS, whereas the majority of normal cells prefer exogenous FAs [40]. Large amounts of studies have shown that knockdown or chemical inhibitors of FASN decrease levels of TGs and PLs and suppresses the growth of cancer cells, even kill cancer cells, while no effects were observed on growth velocity or viability of nonmalignant cells [113, 114]. The cell death induced by FASN inhibitors might result from the toxic accumulation of malonyl-CoA rather than a deficiency of FA [111]. Additionally, targeting FASN can reduce palmitoylation of tubulin and disrupt microtubule organization, thus inhibiting tumor growth [115]. TVB-2640 is the first small molecule compound targeting FASN to enter clinical trials, which can cause partial responses or prolonged overall survival when combined with paclitaxel [116]. Other FASN inhibitors such as C93 and FAS31 have been tested in pre-clinical studies [117]. Nonetheless, severe side effects, including dramatic weight loss and affected cerebral development, impede the clinical application of FASN inhibitors.

Stearoyl-CoA desaturase (SCD) catalyzes the introduction of a *cis* double bond at the C9 position in saturated short-chain FAs to generate monounsaturated fatty acids (MUFAs), such as palmitoleate (C16:1) and oleate (C18:1) from palmitate (C16:0) and stearate (C18:0), respectively [118]. This step alters the physical properties of FAs and has profound impacts on lipid function in tumor cells. There are three isoforms of SCD, including SCD1, SCD5, and fatty acid desaturase 2 (FADS2) identified in humans [118, 119]. The expression and activity of SCD are upregulated in several cancers and are significantly associated with tumor progression and patient outcome [120, 121]. Besides, MUFAs generated by overexpressed SCD are tumor-promoting and essential for chemoresistance and recurrence of tumors [120, 121]. The distinct increased concentrations of MUFAs are noticed in tumor-initiating cells (TICs) of leukemia, OVC, and pancreatic cancer, implying that the desaturated degree of FAs may be a novel TIC marker [122]. Moreover, probably by causing the accumulation of saturated fatty acids, pharmacological inhibition of SCD slows tumor growth down in preclinical cancer models without affecting overall body weight [123]. Recently, sapienate (cis-6-C16:1) biosynthesis catalyzed by FADS2 has been identified as another tumor-promoting FA desaturation pathway, which enables cancer cells to by-pass the desaturation pathway dependent on SCD [119]. This partially can explain why some cancers are insensitive to SCD inhibitors and raises a promising approach of targeting both desaturation pathways for cancer therapy.

Cholesterol is a neutral lipid that is required for the assembly and maintenance of the cell membrane. Increasing evidence suggests the dysregulation of cholesterol biosynthesis is also involved in carcinogenesis and tumor progression [10]. Cholesterol synthesis begins with the conversion of citrate to acetyl-CoA, followed by acetyl-CoA conversion to lanosterol *via* the mevalonate pathway. The rate-limiting step of the mevalonate pathway is catalyzed by HMGCR, which converts HMG-CoA to mevalonate. In addition, proliferating cancer cells frequently exhibit a higher level of HMGCR, leading to increased cholesterol content and higher cholesterol consumption compared to normal proliferating cells [10]. Furthermore, statins, specific HMGCR inhibitors with cholesterol-lowering capacity, reduce the risk of prostate and breast cancer and inhibit the progression of certain cancers [124].

In addition to directly targeting key enzymes, the FAS activities could be repressed by reducing transcription levels. The master transcriptional regulators of lipogenesis contain sterol regulatory element-binding protein 1 (SREBP-1) and carbohydrate-responsive element-binding protein (ChREBP) [125]. SREBP-1 has two isoforms: SREBP-1a and SREBP1c, and SREBP-1c is predominant in most tissues. SREBP-2 mediate cholesterol synthesis, while SREBP-1 plays a central role in cellular FAs and cholesterol biosynthesis *via* regulation of the expression of various lipogenic enzymes, including ACLY, ACC, FASN, SCD, and glycerol-3-phosphate acyltransferase (GPAT) [126]. Hence, inhibiting SREBP-1 suppresses FA synthesis gene expression in cancer cells and probably prevents cancer cell proliferation [127]. Besides, ChREBP regulates gene transcription related to glucose and lipid metabolism *via* binding to carbohydrate response element in the promoter of target genes, such as GLUT2, PKLR, fructokinase, ketohexokinase (KHK), glucose-6-phosphatase catalytic subunit (G6PC), G6PDH, transketolase (TKT), PDK, ACLY, ACC, FASN, and SCD1 et al [128, 129]. Growing evidence indicates that ChREBP plays an essential role in tumorigenesis and progress of cancer; for instance, interference of ChREBP in HCC and CRC cells results in decreased proliferative and tumorigenic potential *in vivo*, accompanied by a metabolic switch from aerobic glycolysis towards OXPHOS, attenuated lipogenesis, and nucleotide synthesis [129]. Taken together, with regard to FAS related enzymes, both SREBP1c and ChREBP regulate expression of ACLY, ACC1, FASN, and SCD1 genes.

After synthesized, to enter the bioactive pool, FAs are covalently modification by CoA *via* ACS [40]. In a bioactive pool, FAs can be esterified with glycerol or sterol backbone to generate TGs or SEs, respectively, and subsequently stored in lipid droplets (LDs). The major TG synthesis pathway is known as the glycerol-phosphate pathway, which condenses FAs with glycerol 3-phosphate *via* using the enzymes GPAT, acylglycerolphosphate acyltransferase (AGPAT), phosphatidic acid phosphohydrolase (PAP), and diacylglycerol acyltransferase (DGAT) in sequence. First, GPAT transfers an acyl group from acyl donor to the sn-1 position of glycerol 3-phosphate. Second, AGPAT esterifies lysophosphatidic acid (LPA) and a FA-CoA to form phosphatidic acid (PA). Third, PAP removes a phosphate group from PA to form diacylglycerol (DG). Next, DGAT enzymes esterify DG and a FA-CoA to form TG. The products of all steps except the last enzyme DGAT can enter into phospholipid synthesis [40]. The major mammalian membrane phospholipid is PC, and multiple cancers display increased PC levels and increased activity of related enzymes in the PC synthesis pathway. Furthermore, knockdown or inhibition of PC synthesis related enzyme can decrease cancer phenotypes [130]. For instance, an inhibitor of choline kinase alpha, the first step of choline activation for PC synthesis, shows benefit for the treatment of advanced solid tumors [131]. Since proliferating cancer cells rely on FAs as cellular building blocks for biosynthesis of membranes and signaling molecules, blocking FAS or lipid biosynthesis holds great perspective as a therapeutic approach for cancer.

### Amino acid synthesis

Amino acids contribute to the majority of biomass synthesis in mammalian cells. Mammals cannot synthesize all the necessary amino acids for protein synthesis and must acquire nine types of amino acids from the diet. While the biosynthesis of the 12 rest of the amino acids, which are classified as NEAAs, is mainly dependent on glutamine metabolism and TCA cycle [19]. Studies show that the consumption of glutamine far exceeds the demands of protein synthesis in cancer cells [132]. Accumulating evidence points that glutamate yielded by glutaminolysis can donate its amine nitrogen towards the biosynthesis of alanine, glycine, serine, proline, and aspartate through a series of enzymatic reactions [19].

Serine and glycine can provide the essential precursors for the synthesis of lipids, proteins, and nucleic acids, as well as affects cellular anti-oxidative ability. Cancer cells produce serine and glycine from glycolysis *via* multi-step reactions [133]. Several enzymes involved in *de novo* serine/glycine biosynthetic pathway including phosphoglycerate dehydrogenase (PHGDH), phosphoserine aminotransferase 1 (PSAT1) and serine hydroxymethyltransferase (SHMT), are highly up-regulated in various cancers and associated with poor-prognosis of cancer patients [134]. Moreover, proline not only serves as an energy source *via* gluconeogenesis of the TCA cycle under stress conditions, but also act as a regulatory substrate for epigenetic reprogramming [135]. Emerging evidence show that proline biosynthesis genes, including pyrroline-5-carboxylate reductase (PYCR1) and aldehyde dehydrogenase 18A1 (ALDH18A1), are poor prognosis factors in different tumor types, suggesting an increased need of proline biosynthesis in cancer [136, 137]. In addition, arginine plays an important role in the regulation of various metabolic and signaling pathways, and high levels of arginine have been observed in multiple types of malignancies [136]. However, the effect of arginine on different cancers is controversial. For some cancers with deficiency of arginine metabolic enzymes such as argininosuccinate synthase 1 (ASS1), arginine deprivation has been considered as a promising strategy [138]. ADI-PEG20, which depletes arginine *via* conversion arginine to citrulline, is an effective inhibitor for several cancers [139]. Taken together, NEAAs generated by glutamine metabolism and the TCA cycle are pivotal for tumor growth, and inhibition of NEAA synthesis is a potential strategy for cancer therapy.

## Catabolic metabolism under metabolic stress

Cancer cells must balance biomass- and energy-producing processes to adapt to the challenging environments, including hypoxia and nutrient deprivation. Tumor cells acquire energy and material basis for rapid tumor growth by enhanced anabolism, while the metastatic tumor cells rely on energy generated by catabolic pathways to survive from metabolic stress during metastasis [11, 13]. Energy is released from the process of ATP hydrolysis to ADP and adenosine monophosphate (AMP), and ATP is generated by aerobic glycolysis, OXPHOS, glutaminolysis, and autophagy in tumor cells [140, 141]. On the one hand, ATP plays a critical role in macromolecule production, DNA synthesis, and membrane integrity [141, 6]. For instance, the synthesis of palmitate from 8 molecules of acetyl-CoA requires seven molecules of ATP and 28 electrons from 14 molecules of NADPH [16]. On the other hand, to survive under stress conditions, including matrix detachment, nutrition deprivation, and chemotherapy, tumor cells must shift their metabolism towards more ATP productivity [142, 143]. For example, ATP depletion in drug-resistant cancer cells leads to chemosensitivity, whereas direct delivery of exogenous ATP renders chemoresistance of drug-sensitive cells [142, 141]. Mounting evidence suggests that cancer cells need higher levels of ATP than normal tissue, and ATP overproduction is closely related to malignancy, invasiveness, chemoresistance, and poor prognosis of cancer patients [142, 143, 141]. Furthermore, the matrix detachment, the first inevitable step of metastasis, quickly causes metabolic stress, resulting from low intracellular ATP level due to decreased activity of GLUTs. Under metabolic stress caused by matrix detachment, essential ATP is generated from autophagy, FAO, and OXPHOS. Therefore, tumor cells must constantly oxidize nutrients to generate sufficient ATP to maintain rapid proliferation and survival under metabolic stress.

### Oxidative phosphorylation

It is well known that for cell proliferation, the bulk of glucose must be diverted to macromolecular precursors such as acetyl-CoA for FAs, glycolytic intermediates for NEAAs, and ribose for nucleotides, only a part of glucose is committed to ATP production [11]. In spite of cancer cells redirect the pyruvate away from TCA by generating lactate, the mitochondrial membrane potential of cancer cells are more active than the normal cells [74]. Mitochondrial ATP is generated from the oxidation of pyruvate, glutamine, FAs, and other respiratory substrates by enzymes of the TCA cycle in the mitochondrial matrix. In detail, respiratory substrates are oxidized in the TCA cycle to generate NADH and FADH2, which are further oxidized in the ETC to generate a proton motive force that comprises of membrane potential (ΔΨ) to drive ATP synthesis [74]. Moreover, during metastasis, peroxisome proliferator-activated receptor-γ coactivator-1α (PGC-1α) mediates mitochondrial biogenesis and OXPHOS to increase ATP production and promote invasion and metastasis [144]. In the glutaminolysis, flux analysis reveals that glutamine is significantly used to replenish TCA cycle intermediates as well as ATP production [34]. To survive under the metabolic stress conditions, the cancer cell can utilize other resources (*e.g.*, lipids, protein, or damaged organelles) to produce essential ATP *via* FAO or autophagy, respectively.

### Fatty acid oxidation

FAs can provide approximately twice as much ATP as glucose and are important energy for cell growth, survival, as well as metastasis when nutrients are limited [104]. Recent research has pointed that FAs derived from TG lipolysis can enter TCA cycle, subsequently produce NADH and FADH2 directly to supply extra ATP [104]. It’s well known that each TG molecule can be hydrolyzed to release three FAs by the sequential function of adipose triglyceride lipase (ATGL), hormone-sensitive lipase (HSL), and monoacylglycerol lipase (MAGL). While most available data for lipases in cancer are related to MAGL, which hydrolyzes the final FA from a monoacylglycerol (MAG), leaving the glycerol backbone. Growing evidence points out that the expression and activity of MAGL are increased in several aggressive cancer cell lines and tumor tissues [145]. Both knockdown and inhibition of MAGL by JZL184 can reduce FAs levels and attenuate pathogenicity of melanoma and OVC cells *in vitro* and *in vivo*, while overexpression of MAGL displays the opposite phenotype [146, 145].

Prior to being oxidized in mitochondrial, FAs are catalyzed to form FA-CoAs, which are subsequently transported from the cytosol across the outer mitochondrial membrane after they are converted to FA carnitines by CPT1 [104]. CPT1 is the first and rate-limiting step of FAO, and it is inhibited by malonyl-CoA, the direct product of ACC2 [109]. CPT1 overexpression in cancer cells promotes FAO and ATP production, adaptation to metabolic stress, and resistance to mTORC1 inhibitors [147]. On the other hand, knockdown and inhibition of CPT1 may deprive ATP and kill cancer cells [147, 148]. In addition, some tumors such as prostate cancer, diffuse large B-cell lymphoma and β-catenin-mutated HCC are highly dependent on FAO for survival and growth [147–149]. Therefore, promoting FAO can increase cellular ATP levels, thus provide energy for cellular proliferation and survival [150, 151].

The nuclear receptor PPARs are pivotal orchestrators of FAO. PPARα regulates FA catabolism by transcriptionally upregulating the expression of many genes, including liver fatty acid-binding protein (L-FABP), CPT1, and medium-chain acyl-CoA dehydrogenase (MADD) genes involved in mitochondrial-oxidation [152]. Additionally, the PPARγ has a wide spectrum of biological functions, including regulating mitochondrial function, energy metabolism, antioxidant defense and redox balance, FAO. Moreover, PPARγ activation by pioglitazone is associated with increased FAO [153, 154]. Therefore, blockade of FAO by targeting PGC-1α/PPARs will be beneficial and potential for reducing the ATP level. Moreover, AMPK, a critical sensor of energy stress, signaled by rising AMP/ATP and ADP/ATP ratios [155]. Activation of the LKB1-AMPK signaling pathway by glucose starvation can increase the catabolic ATP-generating processes, such as FAO and autophagy, and inhibits ATP-consuming biosynthetic processes including protein, cholesterol, and FAs synthesis *via* mTOR inactivation. Activated AMPK phosphorylate and inactivate ACC2 at Ser79, which results in a reduction in malonyl-CoA production, thereby heightening FAO by alleviating the inhibition of malonyl-CoA on CPT1 [155, 156]. On the one hand, AMPK is often downregulated in some tumors, and indeed AMPK activator metformin decreases cancer incidence, suggesting that AMPK may play tumor-suppressive function in carcinogenesis [157]. On the contrary, AMPK activation is closely involved in cancer drug resistance *via* multiple mechanisms such as increased FAO, autophagy induction, and cancer stem cells enrichment [157, 156].

### Autophagy

Autophagy is a lysosome-dependent catabolic process, which includes macroautophagy, microautophagy, and chaperone-mediated autophagy. During the process of autophagy, damaged organelles and misfolded proteins and other autophagic substrates are engulfed in autophagosomes and degraded to simple molecules, such as monosaccharides, FAs, and amino acids [158]. Then, these molecules can be further utilized to produce ATP through catabolic reactions or provide building blocks for the synthesis of essential cellular macromolecules [158]. Besides, autophagosome formation is driven by autophagy-related proteins (ATGs), which form the autophagy activating kinase (UNC-51-like kinase, ULK) complex [158]. The primary functions of autophagy in cancer cells are the regulation of energy metabolism and maintenance of homeostasis, which can enable cells to survive under harsh environments, such as nutrient scarcity, chemotherapies, and irradiation [159, 160]. In detail, first, autophagy synthetically senses the energy state of cells dependent on the energy-sensing cascade kinases in cells include protein kinase A (PKA), AMPK, and mTOR; second, autophagy generates many metabolic substrates as feedback by adjusting the flow of autophagy; and third, autophagy balances ATP consumption and mitochondrial restoration activity to ensure cell survival. However, studies demonstrate that mice deficient for autophagy show promotion in spontaneous tumors [161]; inversely, autophagy-deficient cells display a low ATP level and are more sensitive to target therapies, such as chemotherapy, radiotherapy, and other types of cancer treatments [160]. Additionally, tamoxifen-resistant breast cancer cells could be re-sensitized by the autophagy inhibitor chloroquine (CQ), and resistant to the aromatase inhibitor-exemestane could be reverted by autophagy inhibitors 3-methyladenine (3-MA) and Spautin-1 [162–164].

**Fig. 2:**
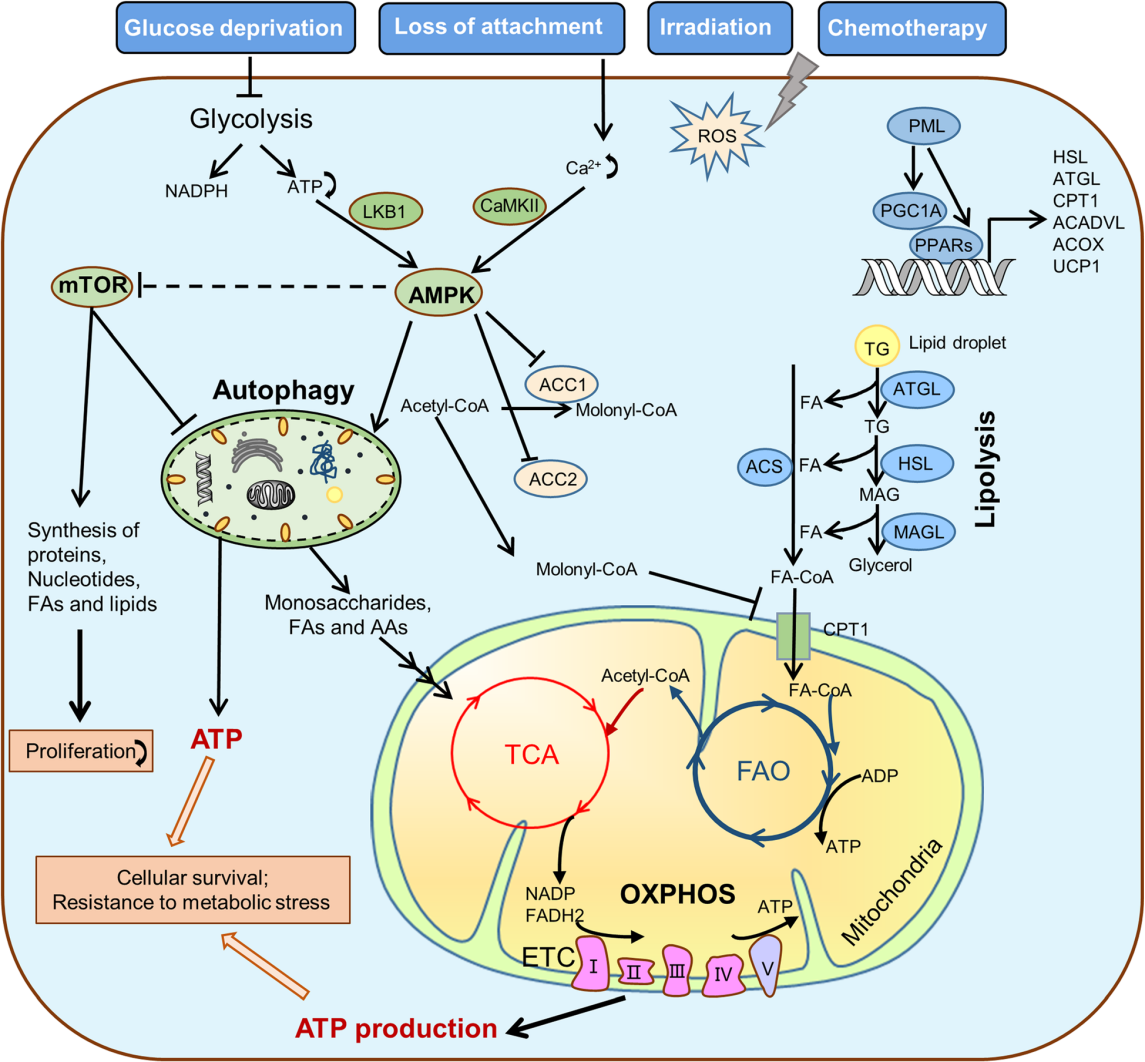
The major catabolic pathways in cancer cells under stress. To survive from stress, such as glucose deprivation, loss of attachment, irradiation or chemotherapy, tumor cells have to reprogram their metabolism from biomass production towards more ATP generation. AMPK, a critical sensor of cellular energy, activated by low ATP level *via* LKB1, or calcium-dependent CaMKII. Once activated, AMPK inhibits mTOR activity, thereby inhibiting synthesis of proteins, nucleotides and lipids, leading to growth arrest. Additionally, AMPK also promotes autophagy by a variety of signal, and autophagy generates many metabolic substrates by breaking down damaged organelles and misfolded proteins, providing small-molecule substrates to TCA cycle and PXPHOS. On the other hand, AMPK inhibits ACC2 activity and reduces the production of molonyl-CoA, which removing the inhibition of molonyl-CoA on CPT1. Furthermore, PML mediates the activation of PGC1-α/PPARs, subsequently, transcriptionally activates the expressions of HSL, ATGL, ACADVL, ACOX2, UCP1 and other genes, promoting lipolysis, FAO, and OXPHOS etc. Moreover, AMPK and PGC1-α/PPARs signaling synergistically facilitate ATP production, maintain the survival and resistance to stress

To survive under stress, such as glucose deprivation, loss of attachment, irradiation, or chemotherapy, tumor cells have to reprogram their metabolism from biomass production towards more ATP generation. Cancer cells activate AMPK, PGC-1a/PPARs, autophagy signaling, then initiate and provide substrates for the TCA cycle, FAO and OXPHOS to produce enough ATP, thus promoting cellular survival (Fig. 2). Therefore, blocking ATP generation will be an attractive approach to improve the therapeutic effect.

## Redox homeostasis

**Fig. 3:**
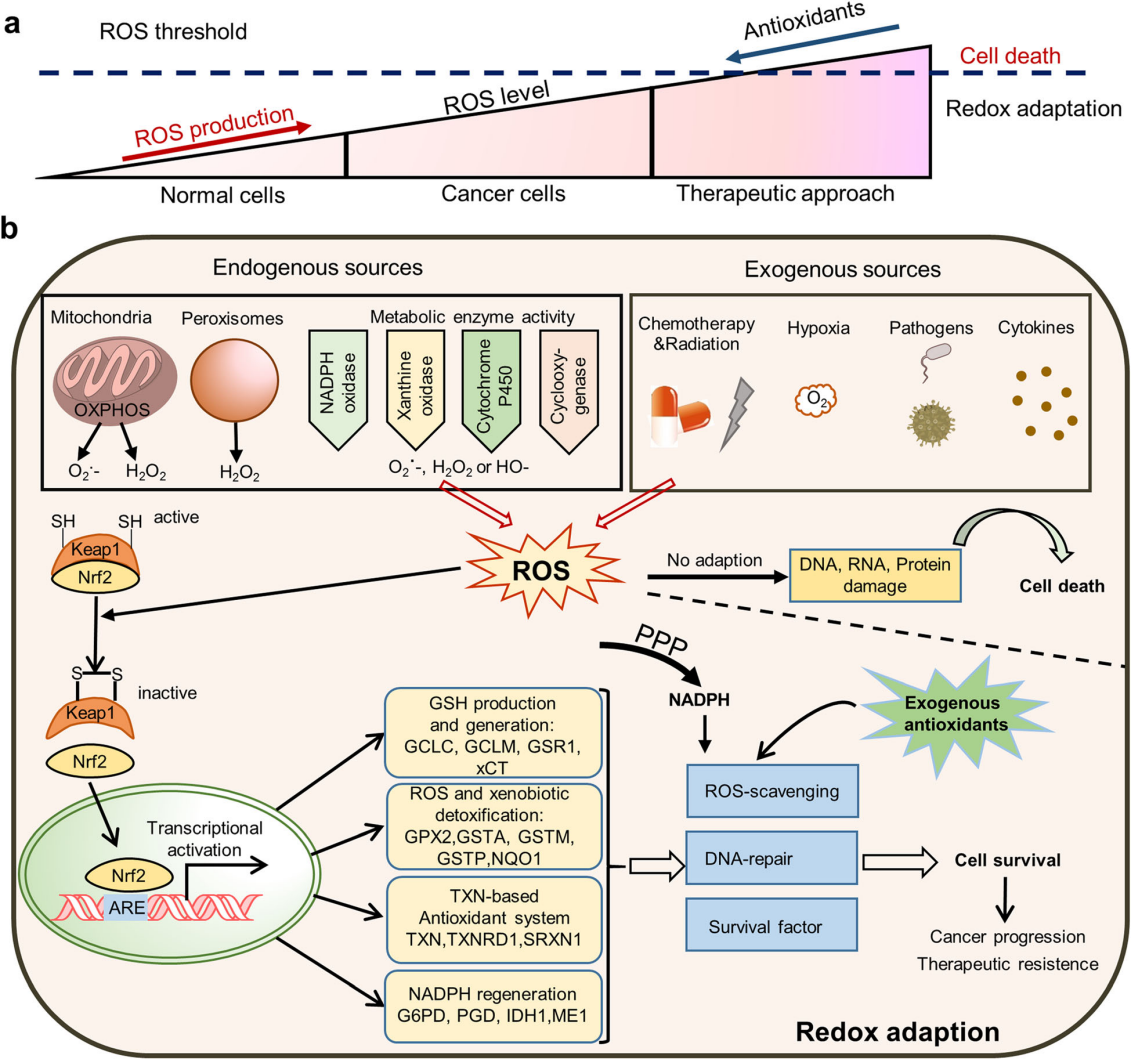
ROS scavenging system in cancer cells. **a** Intracellular redox levels are determined by ROS production and antioxidant activity, and ROS levels of tumor cells were higher than that in normal cells. After treatment, ROS level in cancer cells is further increased. Once the antioxidant capacity of cells fails to eliminate intracellular ROS, high levels of ROS will trigger apoptosis, even cell death. **b** Intracellular ROS generated from both endogenous and exogenous sources. If cancer cells fail to adapt to high level of ROS, intracellular DNA, RNA and proteins will be damaged, even leading to cell death. On the other hand, ROS can activate the antioxidant system of Keap/Nrf2, which activates the expression of a series of downstream antioxidant genes and scavenging ROS. In addition, NADPH generated by the PPP and exogenous antioxidants can also participate in ROS scavenging. Redox adaption promotes cell survival under oxidative stress, and thus facilitating cancer progression and therapeutic resistance

Redox homeostasis is also essential for the maintenance of diverse cellular processes, including cell proliferation and metastasis. ROS, mainly generated in the ETC and NADPH oxidase complex (NOX), are defined as chemically reactive molecules, including superoxide (O_2_-), hydroxyl (OH•), peroxy1 (RO_2_•), and hydroperoxy1 (HO_2_•) radicals [165]. Evidence shows that ROS has double-edged sword characteristics: a mild level of ROS is pro-tumorigenic, while a high level of ROS is cytotoxic [165]. It is well known that proliferative tumor cells possess higher ROS production than normal cells on account of hypermetabolism or tumor microenvironment, while the redox balance is adjusted in tumor cells due to their enhanced antioxidant capacity [166]. Furthermore, therapeutic treatment triggers increased ROS levels in cancer cells. Once the antioxidant capacity of cells fails to reduce intracellular ROS lower than the ROS threshold, high levels of ROS will induce cell death [167] (Fig. 3a).

### Keap1/Nrf2 antioxidant system

It is well known that Nrf2 is the master regulator of the cellular antioxidant response and recognized as a driver of cancer progression, metastasis, and resistance to therapy [168]. Under normal conditions, Nrf2 is rapidly degraded through its interaction with Keap1, nevertheless, under oxidative stress conditions, Keap1 oxidized by ROS leads to Nrf2 activation and translocation to the nucleus, where Nrf2 transactivates a wide spectrum of antioxidant genes *via* binding antioxidant response elements (ARE) on their promoters [169]. Nrf2 regulates the production of glutathione (GSH), the foremost antioxidant molecule within cells, through the expressions of the enzyme that catalyze the rate-limiting reaction of GSH synthesis, including glutamate-cysteine ligase (GCL), glutathione reductase (GSR), and glutathione-S-transferases (GSTs). It also controls free Fe (II) homeostasis *via* activating the expression of heme oxygenase-1 (HO-1), which generates free Fe (II) *via* the breakdown of heme molecules. The NAD(P) H quinone oxidoreductase (NQO1), a ubiquitous cytosolic enzyme that catalyzes the reduction of quinone substrates, is well known as a Nrf2 target (Fig. 3b). The protective role of Nrf2 in the prevention of chemical- and ultraviolet-induced tumorigenesis has been well established [169]. On the other hand, Nrf2 activation in cancer cells accelerates cancer progression and metastasis [170], as well as confers resistance to chemo-and radiotherapy.

### NADPH production

NADPH, a coenzyme that contributes to multiple biological reactions by providing electrons, is essential for scavenging excess ROS and preventing dihydrofolate reductase (DHFR) from degradation in cells [171]. Moreover, rapidly proliferating cancer cells require a sufficient amount of NADH and NADPH for biogenesis such as FAS, and to protect cells from the detrimental effect of ROS [172]. For instance, NADPH produced from the folate pathway contributes to skin cancer cells survival and facilitates metastasis in mouse cancer models [173] and the Snail1-PFKP axis switches glucose flux into PPP and promotes NADPH production, allowing cancer cell survival under metabolic stress, especially in a resource-limited catabolic environment [57, 20].

Intracellular ROS is generated from both endogenous and exogenous sources. Once the antioxidant capacity of cells fails to eliminate intracellular ROS, high levels of ROS induce damaged biological macromolecules and pernicious oxidative stress, which will trigger senescence, apoptosis, ferroptosis, even cell death. On the other hand, ROS can activate the antioxidant system of Keap/Nrf2, forkhead box O (FOXO) as well as PGC1α, which transcriptionally activates the expression of a series of downstream antioxidant genes, thus scavenging ROS. Besides, NADPH generated by the PPP and exogenous antioxidants can also participate in ROS elimination. Hence, the redox adaption promotes cell survival under oxidative stress and thus facilitating cancer progression and therapeutic resistance (Fig. 3b).

## Cancer metabolism remodels the tumor microenvironment

**Fig. 4:**
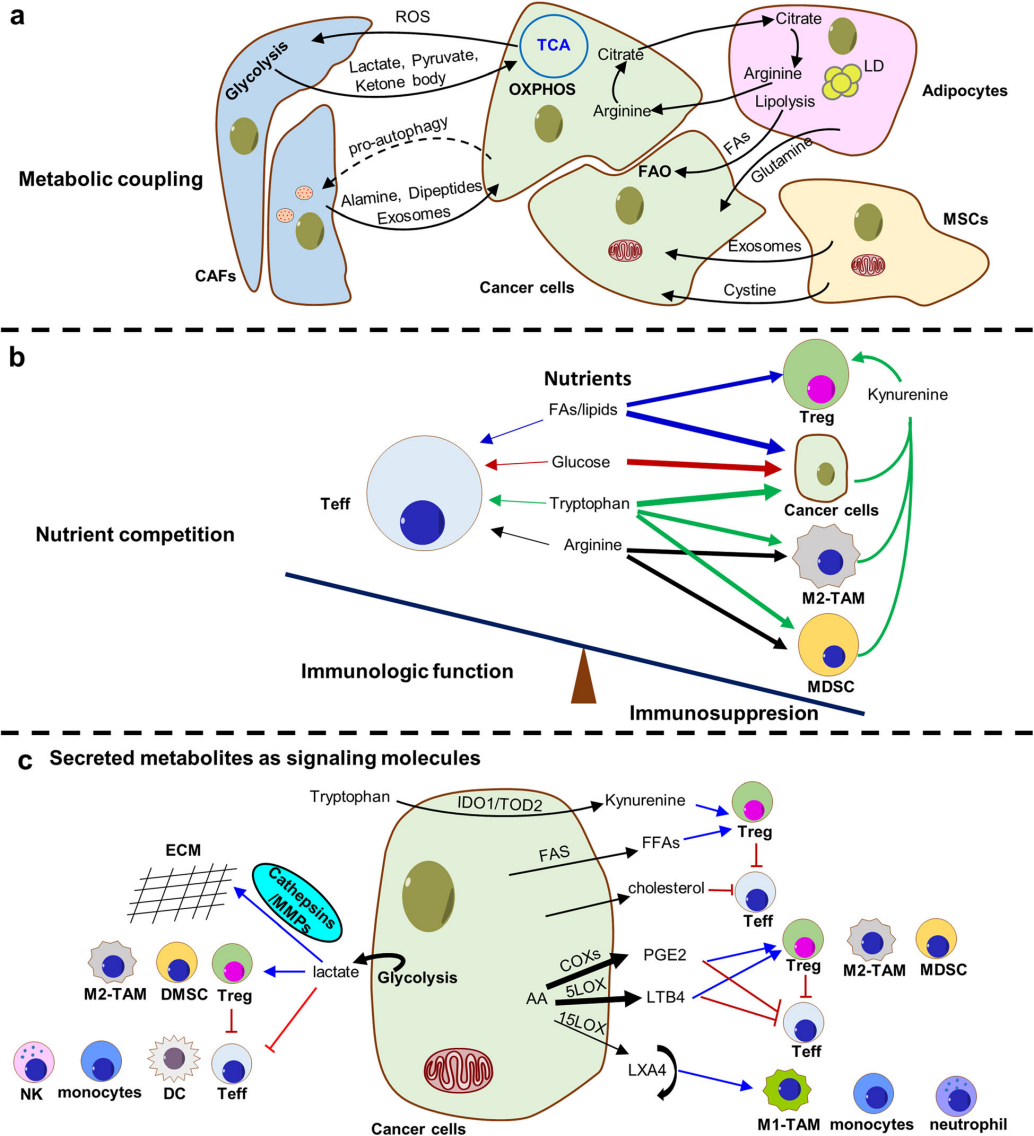
Metabolic interactions in the TME. **a** Metabolic coupling between cancer cells and CAFs, adipocytes, MSCs in tumor environment. **b** Nutrition competition for FAs, glucose, tryptophan and arginine occurring between cancer cells and immunocytes (including Teff, Treg, TAM and MDSC). **c** Cancer cells secrete metabolites as signaling molecules, such as lactate, kynurenine, FAs, cholesterol, PGE_2_, LTB_4_ as well as LXA_4_ to remodel an immunosuppressive TME

Tumor microenvironment (TME) refers to the area surrounding the tumor that is comprised of stromal, immune, and malignant cells, tumor vasculature, and sometimes adipocytes, and the exact composition of each stroma varies depending on cancer and tissue types [174]. TME imposes many challenges for the cancer cells: nutrient deprivation and competition, low pH, oxidative stress, hypoxia, physical pressure, and immune surveillance [175]. However, heterocellular interactions between cancer cells and the TME support tumor growth and immune evasion. Malignant cells adapt TME through symbiotic metabolic interactions with other neighboring cells, including metabolic coupling, nutrient competition, and secreted metabolites as signaling molecules [176] (Fig. 4a-c).

### Metabolic coupling

Cancer-associated fibroblasts (CAFs), a major component of the TME, can become activated by tumor cells, play an important role in carcinogenesis, progression, and metastasis. Recent studies have revealed the metabolic cross-talk between CAFs and tumor cells [177, 178]: tumor cells trigger the “Warburg effect” occurred in the surrounding stromal fibroblasts, promoting the fibroblasts differentiate to CAFs through aerobic glycolysis [179]; CAFs produce the metabolic energy product such as a large number of lactate and pyruvate, which can be taken up by cancer cells; these nutrients can provide fuel for TCA cycle, then produce ATP through OXPHOS pathway, thus promoting tumor cell proliferation and apoptosis resistance. Furthermore, the metabolic coupling between malignant cells and CAFs relies on their unique monocarboxylate transporter (MCT) expression patterns: epithelial tumor cells usually possess high expressions of MCT1, thus promoting the absorption of lactate from the MCT4-expressing CAFs. Besides, acidification induced by lactic acid secreted by CAFs in the local microenvironment can enhance the degradation of extracellular matrix proteins and promote drug-resistance of tumor cells [178, 180]. Conversely, the metabolic coupling between cancer cells and stromal fibroblasts also may occur in an opposite pattern. For instance, metabolic symbiosis has been described between glycolytic cancer cells and oxidative stromal fibroblasts in a colorectal cancer model [178]. Additionally, in response to tumor-derived factors, adipocytes release free FAs through lipolysis, which can be directly taken up and utilized by cancer cells to sustain the tumor growth *via* β-oxidation [43, 44, 181]. Besides, in a nutrient-poor microenvironment, pancreatic ductal adenocarcinoma cells promote autophagy of pancreatic stellate cells (PSCs), and autophagic PSCs release alanine, which is captured by the pancreatic cancer cells and used to fuel macromolecular biosynthesis [182]. Therefore, the metabolic symbiosis between cancer cells and stromal cells promotes tumor growth and metastasis.

### Nutrient competition

It is clear that tumor cells have a stronger capability to obtain nutrients, and nutrient deficiency is an important characteristic of TME [12]. Studies suggest that altered energy metabolism in tumor-associated macrophages (TAM) can cause distinct polarization states: M1 (antitumor) macrophages prefer glycolysis, while M2 macrophages (protumor) predominantly dependent on OXPHOS and FAO. Furthermore, tumor-infiltrating macrophages are usually M2-type and are associated with glucose deprivation in the TME [183]. Besides, glucose is critical to T-cell proliferation and effector function, and effective immune response of T-cell relies on glucose uptake *via* GLUT1. Furthermore, cancer cells can also attenuate antitumor T-cell responses by competing for available glucose with cytotoxic tumor-infiltrating lymphocytes (TILs), creating an immunosuppressive environment through TIL starvation [184]. In a sarcoma model, T cell responses are restored by blocking glycolysis in cancer cells because more glucose is then available for the TILs [185].

In addition, arginine metabolism also plays an important role in T cell activation and immune regulation [185]. Arginine in TME is rapidly catabolized by immunocyte expressing arginase 1 (ARG1), like myeloid-derived suppressor cells (MDSCs), regulatory T cells (Tregs), M2-macrophages, creating a state of arginine deficiency and limiting the availability of T cells to arginine, which suppresses antitumor immunity [186]. Studies have shown that arginine supply activates the cytotoxicity of T and NK cells as well as the production of effector cytokines, and combination of arginine and PD-L1 antibody significantly enhanced the anti-tumor immune response and extended the survival time of mice model with osteosarcoma [187]. Furthermore, tumor-infiltrating Tregs take up more FAs from TME than other TILs, which further enhance the accumulation of Treg cells to promote an immunosuppressive microenvironment. Hence, this nutrient competition in the tumor microenvironment impairs effective antitumor immunity, which facilitates tumor growth.

### Secreted metabolites as signaling molecules

Beyond uptake nutrients with avarice, tumor cells also secrete metabolites that act as signaling molecules, thus remodel the TME. Due to increased glycolysis, a large amount of lactate is produced and then exported to extracellular by MCTs, thus forming an acidic microenvironment. Both hypoxia and low pH are well-established features of the tumor microenvironment that may encourage tumorigenesis by suppressing antitumor immunity [188]. The acidification in the TME is conducive to the activation of certain proteases, including matrix metalloproteinases (MMPs), urokinase-type fibrinogen, and histoproteases, as well as inducing the degradation of extracellular matrix (ECM), thus promoting the metastasis of tumor cells [189]. Moreover, high levels of lactate released by cancer cells not only inhibits the proliferation and function of TILs but also drive T-cells towards a Treg phenotype, which promote immunosuppressive microenvironment [190].

Additionally, metabolites of arachidonic acid (AA), such as prostaglandins (PGs), leukotrienes (LTs), and lipoxins (LXs), have extensive physiological functions and play important roles in the regulation of inflammation, immune and cancer [191]. It is possible that normal cells utilize AA, predominantly to generate LXA_4_ (and resolvins, protectins, and maresins), whereas tumor cells use them to produce mainly PGE_2_ and LTs. PGE_2_, a derivative of AA, is produced by cyclooxygenases (COX; constitutive COX1 and inducible COX2) and PG synthase in many tumor cells and then secreted to extracellular. Moreover, PGE_2_ in the TME elicits a wide range of biological effects associated with immunopathology in inflammation and cancer; for instance, PGE_2_ excretion from tumor cells is critical for M2-type polarization and infiltration of macrophage [192]; PGE_2_ can suppress the activation of CD4^+^ and CD8^+^ T cells and leads to dysfunction of T cells [193], and PGE_2_ derived from CRC and MDSCs exacerbates the immunosuppressive activity of MDSCs and accelerated tumor growth of CRC [194]. Additionally, upregulated 5-lipoxygenase expression and metabolites (*e.g.,* 5-HETE and LTB_4_) production are observed in a number of cancer types and have been shown to be associated with immunosuppressive TME and increased tumorigenesis [195]. However, LXA_4_, considered as “braking signals” of inflammation, is markedly reduced in multiple cancers, including CRC, HCC, and OVC [196]. Some evidences show that LXA^4^ exerts anti-inflammatory effects in TME, leading to tumor cell apoptosis [197, 196]. In tumors, certain beneficial, even anticancer, metabolic components are significantly reduced, such as lipoxins and resolvins. Supplement with these metabolites or enhancing the synthesis of beneficial metabolites pathways may be beneficial to the prevention and treatment of tumors [198]. In addition, treatment with LXA_4_ at an early stage of the tumor, could significantly inhibit tumor growth in a variety of tumor models [199, 197]. Omega-3 polyunsaturated fatty acids (ω-3 PUFAs) include α-linolenic acid (ALA; 18:3 ω-3), stearidonic acid (SDA; 18:4 ω-3), eicosapentaenoic acid (EPA; 20:5 ω-3), docosapentaenoic acid (DPA; 22:5 ω-3), and docosahexaenoic acid (DHA; 22:6 ω-3), are applied for chemoprevention or adjuvant therapy of gastrointestinal cancers [200]. Through a series of reactions containing 15-lipoxygenase (15-LOX), AA can produce endogenous specialized pro-resolving lipid mediators (SPMs), named lipoxins, which are stop-and-go signals for the inflammation resolution phase. Similarly, resolvins derived from EPA, DHA, and DPA, as well as maresins and protectins are DHA- and DPA-derived SPMs [200]. These SPMs possess the ability to promote inflammation regression and inhibiting the progression of cancers [201]. Therefore, dietary supplements of ω-3 PUMAs show great benefit for chemoprevention or adjuvant therapy of multiple cancers.

Besides, in gastric cancer (GC) cells, RHOA-Y42 mutations activate the PI3K-AKT-mTOR pathway, then increase the production of FAs that are more effectively consumed by Treg cells than cytotoxic T lymphocyte (CTL), generating an immunosuppressive TME that underlies resistance to immune checkpoint blockade [175]. Under physiological conditions, kynurenine generated from tryptophan catabolism is crucial for the maintenance of placental immune privilege. However, tryptophan metabolism is utilized by malignant cells to confront the immune system in many tumors [202]. Tryptophan is identified as an endogenous aryl hydrocarbon receptor (AHR) ligand, and activation of AHR signaling in primed CD4^+ ^T cell differentiation towards the immunosuppressive Treg phenotype and suppresses dendritic cell immunogenicity. Indoleamine­2, 3­dioxygenase (IDO), the rate­limiting enzyme of tryptophan catabolism, is highly expressed in multiple types of cancer cells and in intratumoral antigen­presenting cells [203]. Recently, IDO inhibitors (such as indoximod and epacadostat) are being tested in clinical trials as immunotherapies for cancer patients, aiming to enhance the efficacy of other immunotherapy (including PD-1 and CTLA-4 inhibitors) [204]. Taken together, these studies indicate that these metabolites generated from tumor cells participate in the formation of the immunosuppressive microenvironment.

## Therapy targeting tumor metabolism

Chemotherapies targeting metabolism have been shown effective in cancer treatments in clinics, and the success of these therapies indicates that a therapeutic window exists to target the metabolism of cancer [205, 28]. Here, we summarize some compounds that targeting tumor metabolism, including preclinical, in the trail, and approved (Table 2). However, there are still many limitations of this metabolic intervention strategy, such as chemotherapy resistance and severe side effects, due to metabolic heterogeneity in tumors, the plasticity of metabolic pathways, some metabolic targets are undruggable, as well as certain metabolic enzymes are universally expressed in the body. In this section, we highlight the potential approach of metabolic targets for cancer therapies.

**Table 2:** Targeting intervention for tumor metabolism (In preclinical, in trials or approved)

Classification	Name	Target enzyme	Status
Glucose uptake inhibitors	Phloretin	sodium/glucose cotransporters	In trials
	Fasentin	GLUT1/4	In trials
	WZB117	GLUT1	In trials
	STF-31	GLUT1	In trials
	BAY-876	GLUT1	In trials
FAs/Lipids uptake inhibitors	Lipofermata	FATP	In preclinical
	Ursodiol	FATP5	In trials
Glutamine uptake inhibitors	V-9302	ACST2	In preclinical
Glycolisis pathway inhibitors	2-DG	HK2	In trials
	3-BP	HK2	In trials
	Lonidamine	HKs, MPC, plasma membrane monocarboxylate transporters	In trials
	PDK4-IN-1	PDK4	In trials
	GSK2334470	PDK1	In preclinical
	PS210	PDK1	In preclinical
	VER-246608	PDHK	In preclinical
	UK-5099	mitochondirial pyruvatecarrier (MPC)	In preclinical
	Sodium dichloroacetate	PDHK	In trials
	FX-11	LDHA	In preclinical
	GNE-140 racemate	LDHA	In preclinical
	GSK2837808A	LDHA	In preclinical
	(R)-GNE-140	LDHA	In preclinical
	LDH-IN-1	LDHA	In preclinical
PPP inhibitor	6-AN	G6PDH	In preclinical
TCA cycle mediators	AG-120	mutant IDH1	In trials
	IDH305	mutant IDH1	In trials
	BAY1436032	mutant IDH1	In trials
	FT-2102	mutant IDH1	In trials
	AG-221	mutant IDH2	In trials
	AG-881	mutant IDH1、2	In trials
	CPI-613	(PDH)/a-KG dehydrogenase	In trials
	Compound 7	MDH2	In preclinical
Nucleotide synthesis inhibitors	5- fluorouracil	thymidylate synthase	Approved
	Capecitabine	thymidylate synthase	Approved
	Methotrexate	dihydrofolate reductase	Approved
Glutaminolysis inhibitors	V-9302	ACST2	In preclinical
	CB-839	GLS	In trials
	C-968	glutaminase C (GAC)	In trials
	BPTES	glutaminase	In trials
	AOA	aminotransferase	In trials
FAS inhibitors	C75	FASN	In preclinical
	TVB-3166	FASN	In preclinical
	C93	FASN	In preclinical
	FAS31	FASN	In preclinical
	TOFA	ACC1	In preclinical
	MK-4074	ACC	In preclinical
	BTA	CIC	In preclinical
	MK-8245	SCD	In preclinical
	GSK1940029	SCD	In preclinical
Cholesterol synthesis inhibitors	Pitavastatin Calcium	HMG-CoA	In trials
	Rosuvastatin Calcium	HMG-CoA	In trials
	Avasimibe	acyl coenzyme A-cholesterol acyltransferase (ACAT)	In trials
Lipid signaling molecules synthesis inhibitors	Aristolochic acid C	phospholipase A2	In preclinical
	Rilapladib	phospholipase A2	In preclinical
	Aspirin	COX1/2	Approved
	Celecoxib	COX2	Approved
	DG051	Aminopeptidase	In trials
	TK05	gutathione S-transferase	In preclinical
NEAA synthesis inhibitors	ADI-PEG20	argininosuccinate synthase	In trials
ETC inhibitors	Metformin	mitochondrial complex I	In trials
	Rotenone	mitochondrial complex I	In preclinical
	Bullatacin	mitochondrial complex I	In preclinical
	α-TOS	mitochondrial complex II	In preclinical
	Benzylisothiocyanate	mitochondrial complex III	In preclinical
Lipolysis inhibitors	JZL184	MAGL	In preclinical
	Atglistatin	ATGL	In preclinical
FAO inhibitors	Etomoxir	CPT1α	In trials
	Perhexiline	CPT1α	In trials
	ST1326	CPT1α	In trials
Autophagy inhibitors	chloroquine	autophagy	In trials
	3-MA	autophagy	In preclinical
	Spautin-1	autophagy	In preclinical
IDO panthway inhibitors	Indoximod	IDO	In trials
	Epacadostat	IDO	In trials
Specialized pro-resolving mediators	LXA_4_	formyl peptide receptor like 1 (FPRL1)	In preclinical
	Resolvin D1	Resolvin D1 receptor	In preclinical

### Combination of inhibitors targeting different metabolic pathways

At present, the effect of single signaling pathway targeted therapy is not satisfactory in clinics, due to the metabolic heterogeneity in tumors [8] as well as the activation of alternative metabolic pathways [206]. To overcome the drug resistance caused by metabolic heterogeneity or plasticity, combining multiple metabolic pathway inhibitors is usually adopted. For instance, glioblastoma cell subpopulations with distinct metabolic requirements: the fast-cycling rely on aerobic glycolysis and sensitive to glucose deprivation or glycolysis inhibition with 2-DG; while slow-cycling cells preferentially utilize OXPHOS for their functions and sensitive to pharmacological inhibition of the ETC, OXPHOS, as well as FABP7 [207]. Survival is significantly improved when glioblastoma tumors are treated with FABP7 inhibition combination with 2-DG [207]. Moreover, tumor cells possess metabolic flexibility; after blocking the primary metabolic pathway, tumor cells will launch the alternative metabolic pathways to maintain their growth and survival. If the primary and alternative metabolic pathways are blocked; meanwhile, the therapeutic effect will be significantly improved. For example, blocking anaerobic glycolysis will activate the PPP pathway, and the combination of 2-DG (an inhibitor of HK2) and 6-AN (an inhibitor of G6PDH) has been shown to enhanced radiation-induced damage in glioma and squamous carcinoma cells [208]. Cancers in different types or states may depend on distinct metabolic pathways. Therefore, the identification of tumor-specific metabolic pathways has become the focus and difficulty in the research of tumor metabolism.

### Induction of timed metabolic collapse

According to evolutionary biology, when a group suffers a stressful event, or "bottleneck," only adaptive individuals can survive, which explains the clonal evolution of cancer [209]. AML is a highly lethal cancer of the hematopoietic system in which residual cloning persists through treatment and eventually leads to recurrence [210]. In response to chemotherapy, AML cells exhibit transient metabolic changes with enhanced glutamine or pyrimidine metabolism, which drive the resistance to chemotherapy [211]. Moreover, blocking glutamine metabolism or pyrimidine synthesis can select eliminating residual leukemia-initiating cells and improve overall survival in leukemia mouse models and patient-derived xenografts [211], suggesting that timed cell-intrinsic or niche-focused metabolic interference induce a metabolic collapse in cancer cells to overcome chemoresistance.

### Metabolic editing of CAR T-cells

Cytotoxic T cells rely on microenvironment nutrients to proliferate and function, and within the mission to destroy tumor cells. At the same time, tumor cells display a stronger capability to obtain nutrients, leading to a nutrient-deficient microenvironment, which causes T cell exhaustion [184]. Nowadays, how to enhance the function of T cells so that they can effectively immunize malignant tumor cells is pivotal for tumor immunotherapy. Studies show that, compared with non-responders, CD8^+^ CAR T cells have enhanced mitochondrial biogenesis in complete responding chronic lymphocytic leukemia patients, which is positively related to the expansion and persistence of CAR T cells [212]. Additionally, bezafibrate, an agonist of PGC-1α/PPAR complexes, activates CTL mitochondria and upregulates OXPHOS as well as glycolysis, enhancing the proliferation of naive T cells and function in CTLs [153] Furthermore, enhanced PPAR-α signaling and FAO can partially preserve CD8^+^ T cell functions when subjected to hypoglycemia and hypoxia [213]. Hence, improving the metabolic capacity of CAR-T cells by the introduction of transcription factors (*e.g.*, PPARα and PGC-1α) or treatment of agonist of PGC-1α/PPAR signaling will promote the function of CAR-T cells in some solid tumors.

### Correcting the mutation of metabolic enzymes by CRISPR/Cas9

So far, a total of eight mutated metabolic genes have been identified in tumors. These eight genes are FH, SDHA, SDHB, SDHC, SDHD, SDHAF2, IDH1, and IDH2 respectively [94, 76]. Interestingly, all of these genes encode key enzymes in the TCA cycle. However, it is extremely difficult to develop corresponding targeted drugs. Application of the CRISPR/Cas9 system to correct the mutation at the coding region of key enzymes is a promising approach for cancer therapy [214, 215]. However, as this technology is still in its early stage, further investigation and improvements are needed to ensure its accuracy and safety. Additionally, improving the balance of efficient DNA editing and inhibition of potential oncogenic effects is also important.

## Conclusion

If tumor cells are likened to “criminals,” the immunocytes are the “policemen,” and the other neighboring cells are the “onlookers.” On the one hand, the “criminal” frantically grab energy sources (*e.g.*, glucose, fatty acids, lipids, and glutamine et al.) from environment [23–25]; and then produce biomass to fulfill their requirements *via* enhanced synthetic pathways, including aerobic glycolysis, glutaminolysis, FAS and PPP [11]. On the other hand, ATP and NADPH, just like money, as the currency of energy and redox balance of “criminal,” is indispensable to maintain the survival, proliferation, redox homeostasis, and metastasis of tumor cells [142, 141, 21, 57]. Through metabolic reprogramming, the “criminals” remodel the microenvironment, leading to the involvement of "policemen" and "onlookers" in their criminal activities [216, 176]. In the above contents, we reviewed the metabolic activity patterns of the “criminals” and their complex metabolic relationships between the “policemen” and “onlookers”, and summarized current intervention strategies, aiming to provide theoretical support for the precise eliminating the “criminals”-tumor cells.

At present, the use of PET-CT, high-dose of vitamin C, metformin, dichloroacetic acid, and ketogenic diet have proved that intervention in tumor metabolism can effectively prevent and inhibit certain tumors. However, the field of tumor metabolism is still facing many new challenges. (1) Because enzymes in metabolic pathways tend to have multiple isoforms, small molecule inhibitors may fail to distinguish between the isoforms expressed in tumor cells and normal cells, for example, COX-1 and COX-2 [217]. Even if specific inhibitors are developed, drug-resistance may occur during treatment due to metabolic compensation of other subtypes of enzymes. (2) Due to the plasticity of metabolic pathways, allow tumors to quickly switch adaptation mechanisms in the face of stress [206]. In order to avoid adaptive resistance of tumor cells, the alternative pathway activated by metabolic interventions should be suppressed in clinics. (3) Heterogeneity, including metabolic heterogeneity, brings great difficulty to treat cancer. Inhibition of a single metabolic pathway may result in treatment failure because the subpopulations insensitive to the inhibition will rapidly proliferate. (4) These tumors are driven by some gene mutations or amplification, such as C-Myc amplification and FH mutations. There are no direct targeted drugs, or it is extremely difficult to develop corresponding targeted drugs. Notwithstanding many other deficiencies, the application of the CRISPR/Cas9 system to DNA correcting the key enzymes is a potential strategy for anti-cancer therapy. (5) Despite displaying significant inhibitory effects on tumor cells *in vitro*, many applications of metabolic regulators in clinics are limited due to their invalidity in the body or severely side effects [218]. Cancer cells share the same metabolic pathways as normal cells, therefore making it difficult to intervene in tumor metabolism without affecting normal cells. At present, our relevant studies on tumor metabolism cannot accurately simulate the energy metabolism occurring in the TME. Models that can better mimic the TME in cancer patients need to be established.

In conclusion, various drugs targeting metabolism with high efficacy are applied for particular diseases, but more metabolic therapies are limited due to severe side effects. Now, targeting tumor metabolism remains as an attractive anticancer therapy because the metabolic heterogeneity within and between tumors is much less than the genetic heterogeneity of tumors. Furthermore, a better understanding of the metabolic heterogeneity in specific tumor tissues and structural information of rate-limiting enzyme holds the key for seeking “Achilles' heel” for tumor growth and utilizing those weaknesses for better cancer therapy.

## Abbreviations

15-LOX: 15-lipoxygenase
2-DG: 2-deoxyglucose
2-HG: 2-hydroxyglutarate
3-BP: 3-bromopyruvate
3-MA: 3-methyladenine
6-AN: 6-aminonicotinamide
6PGDH: 6-phosphogluconate dehydrogenase
6-PGL: 6-phosphogluconolactonase
α-KG: α-ketoglutarate
α-KGDDs: α-KG dependent dioxygenases
AA: arachidonic acid
ACC: acetyl-CoA carboxylase
ACLY: ATP citrate lyase
ACS: acyl-CoA synthetases
ADP: adenosine diphosphate
AGPAT: acylglycerolphosphate acyltransferase
AH: aconitate hydratase
AHR: aryl hydrocarbon receptor
ALA: α-linolenic acid
ALDH: aldehyde dehydrogenase
ALL: acute lymphoblastic leukemia
ALT: alanine transaminase
AML: acute myeloid eukemia
AMP: adenosine monophosphate
AMPK: AMP-activated protein kinase
ARE: antioxidant response elements
ARG1: argininase 1
ASCT2: alanine, serine, cysteine–preferring transporter 2
ASS1: argininosuccinate synthase 1
AST: aspartate transaminase
ATGL: adipose triglyceride lipase
ATGs: autophagy-related proteins
ATP: adenosine triphosphate
BTA: benzene-tricarboxylate analog
CAFs: cancer-associated fibroblasts
CAR-T: chimeric antigen receptor T cells
ChREBP: carbohydrate-responsive element-binding protein
CIC: citrate carrier
COX: cyclooxygenases
CPT: carnitine palmitoyl transferase
CQ: chloroquine
CRC: colorectal cancer
CREB: cAMP response element-binding protein
CS: citrate synthase
CSCs: cancer stem cells
CTL: cytotoxic T lymphocyte
DCA: 2-chloroacetic acid
DG: diacylglycerol
DGAT: diacylglycerol acyltransferase
DHA: docosahexaenoic acid
DHEA: dehydroepiandrosterone
DHFR: dihydrofolate reductase
DPA: docosapentaenoic acid
ECM: extracellular matrix
EPA: eicosapentaenoic acid
ESCC: esophageal squamous cell carcinoma
ETC: electron transport chain
F-1,6-BP: fructose-1,6-bisphosphate
F-6-P: fructose-6-phosphate
FA: fatty acid
FABP: fatty acid binding proteins
FADS2: fatty acid desaturase 2
FAO: fatty acid oxidation
FAS: fatty acid synthesis
FASN: fatty acid synthase
FATP: fatty acid transport proteins
FDG/PET: fluorodeoxyglucose positron emission tomography
FH: fumarate hydratase
FOXM1: forkhead box M1
FOXO: forkhead box O
G-3-P: glyceraldehyde-3-phosphate
G-6-P: glucose-6-phosphate
G6PC: glucose-6-phosphatase catalytic subunit
G6PDH: glucose-6 phosphate dehydrogenase
GC: gastric cancer
GCL: glutamate-cysteine ligase
GIST: gastrointestinal stromal tumors
GLS: glutamine synthetase
GLUD: glutamate dehydrogenase
GLUT: glucose transporter
GPAT: glycerol-3-phosphate acyltransferase
GSH: glutathione
GSR: glutathione reductase
GST: glutathione-S-transferases
HCC: hepatocellular carcinoma
HIF1-α: hypoxia inducible factor-1A
HK2: hexokinase 2
HLRCC: hereditary leiomyomatosis and renal cell cancer
HMG-CoA: 3-hydroxy-3-methylglutaryl-coenzyme A
HMGCR: hydroxy-3-methyl glutaryl coenzyme A reductase
HO-1: heme oxygenase-1
HO_2_-: hydroperoxy
hPGLs: hereditary paragangliomas
HSL: hormone sensitive lipase
ICB: immune checkpoint inhibitors
IDH: isocitrate dehydrogenase
IDO: Indoleamine­2, 3­dioxygenase
Keap1: Kelch-like ECH-associated protein 1
KHK: ketohexokinase
KLF4: krüppel-like factor 4
LAT1: large neutral amino acid transporter 1
LDHA: Lactate dehydrogenase A
LDs: lipid droplets
LPA: lysophosphatidic acid
LTs: leukotrienes
LXs: lipoxins
MADD: medium-chain acyl-CoA dehydrogenase
MAG: monoacylglycerol
MAGL: monoacylglycerol lipase
MCT: monocarboxylate transporter
MCUL: uterine leiomyomas
MDH: malate dehydrogenase
MDSCs: myeloid-derived suppressor cells
MMPs: matrix metalloproteinases
MUFAs: monounsaturated fatty acids
NADPH: nicotinamide adenine dinucleotide phosphate
NEAAs: non-essential amino acids
NOX: NADPH oxidase complex
NQO1: NAD(P) H quinone oxidoreductase
Nrf2: nuclear factor erythroid 2-related factor
NSCLC: non-small cell lung carcinomas
O_2_-: superoxide
OAA: oxaloacetate
OH•: hydroxyl
OVC: ovarian cancer
OXPHOS: oxidative phosphorylation
PA: phosphatidic acid
PAP: phosphatidic acid phosphohydrolase
PCCs: pheochromocytomas
PDG-PET: fluorodeoxyglucose positron emission tomography
PDH: pyruvate dehydrogenase complex
PDK: pyruvate dehydrogenase kinase
PE: phosphatidyl ethanolamine
PEP: phosphoenolpyruvate
PFK: phosphofructosase
PG: prostaglandin
PGC-1α: peroxlsome proliferator-activated receptor-γ coactlvator-1α
PHGDH: phosphoglycerate dehydrogenase
PKA: protein kinase A
PKM2: pyruvate kinase M2
PL: phospholipid
PPAR: peroxisome proliferator-activated receptors
PPP: pentose phosphate pathway
PRODH: proline dehydrogenase
PSAT1: phosphoserine aminotransferase 1
PSCs: pancreatic stellate cells
PSPH: phosphoserine phosphatase
PYCR1: pyrroline-5-carboxylate reductase
R5P: ribose-5-phosphate
RO_2_•: peroxy
ROS: reactive oxygen species
Ru5P: riburose-5-phosphate
SCD: stearoyl-CoA desaturase
SDA: stearidonic acid
SDH: succinate dehydrogenase
SEs: sterol esters
SHMT: serine hydroxymethyltransferase
SPMs: pro-resolving lipid mediators
SREBP-1: sterol regulatory element-binding protein 1
TAM: tumor-associated macrophages
TCA: tricarboxylic acid
TGs: triglycerides
TICs: tumor-initiating cells
TILs: tumor-infiltrating lymphocytes
TKT: transketolase
TME: tumor microenvironment
ULK: UNC-51-like kinase
